# Structural basis for a highly conserved RNA-mediated enteroviral genome replication

**DOI:** 10.1093/nar/gkae627

**Published:** 2024-07-22

**Authors:** Naba Krishna Das, Jeff Vogt, Alisha Patel, Hasan Al Banna, Deepak Koirala

**Affiliations:** Department of Chemistry and Biochemistry, University of Maryland, Baltimore County, Baltimore, MD 21250, USA; Department of Chemistry and Biochemistry, University of Maryland, Baltimore County, Baltimore, MD 21250, USA; Department of Chemistry and Biochemistry, University of Maryland, Baltimore County, Baltimore, MD 21250, USA; Department of Chemistry and Biochemistry, University of Maryland, Baltimore County, Baltimore, MD 21250, USA; Department of Chemistry and Biochemistry, University of Maryland, Baltimore County, Baltimore, MD 21250, USA

## Abstract

Enteroviruses contain conserved RNA structures at the extreme 5′ end of their genomes that recruit essential proteins 3CD and PCBP2 to promote genome replication. However, the high-resolution structures and mechanisms of these replication-linked RNAs (REPLRs) are limited. Here, we determined the crystal structures of the coxsackievirus B3 and rhinoviruses B14 and C15 REPLRs at 1.54, 2.2 and 2.54 Å resolution, revealing a highly conserved H-type four-way junction fold with co-axially stacked sA-sD and sB-sC helices that are stabilized by a long-range A•C•U base-triple. Such conserved features observed in the crystal structures also allowed us to predict the models of several other enteroviral REPLRs using homology modeling, which generated models almost identical to the experimentally determined structures. Moreover, our structure-guided binding studies with recombinantly purified full-length human PCBP2 showed that two previously proposed binding sites, the sB-loop and 3′ spacer, reside proximally and bind a single PCBP2. Additionally, the DNA oligos complementary to the 3′ spacer, the high-affinity PCBP2 binding site, abrogated its interactions with enteroviral REPLRs, suggesting the critical roles of this single-stranded region in recruiting PCBP2 for enteroviral genome replication and illuminating the promising prospects of developing therapeutics against enteroviral infections targeting this replication platform.

## Graphical Abstract

**Graphical Abstract:**
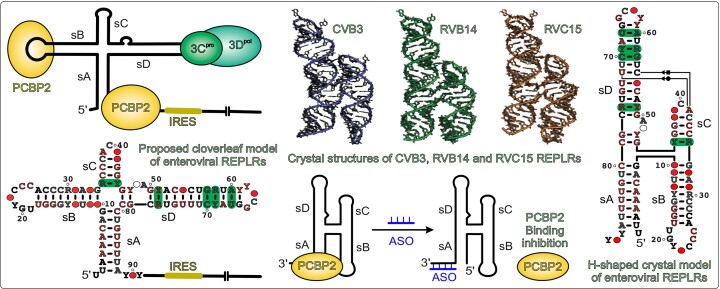

## Introduction

The *enterovirus* genus within the *Picornaviridae* family includes RNA viruses responsible for several human diseases, such as the common cold, poliomyelitis, acute flaccid paralysis, and myocarditis (1–3). A typical enterovirus encapsulates a (+)-strand RNA genome of about 7.5 kb length, which is polyadenylated at the 3′ end and linked covalently with the viral protein VPg at the 5′-end (4–7). The genomic RNA, which also serves as mRNA, harbors several RNA structures at the 5′ and 3′ untranslated regions (UTRs) to promote and regulate the genome replication and genome-encoded protein translation. Specifically, the 5′ UTR contains several modular RNA domains, designated I to VII (Supplementary Figure S1), that recruit the viral and host proteins to form functional ribonucleoprotein (RNP) complexes through various noncanonical mechanisms. Whereas the domains II to VII immediately upstream of the open reading frame (ORF) represent internal ribosome entry site (IRES) elements responsible for viral genome translation (8–11), the domain I at the extreme 5′ end constitutes an essential platform that assembles an RNP complex required for the replication initiation (12–15). In this study, we have determined the structural basis for domain I RNA-mediated enteroviral genome replication using representative genotypes from the seven species, including a genotype from enterovirus A to D and rhinovirus A to C.

**Figure 1.**
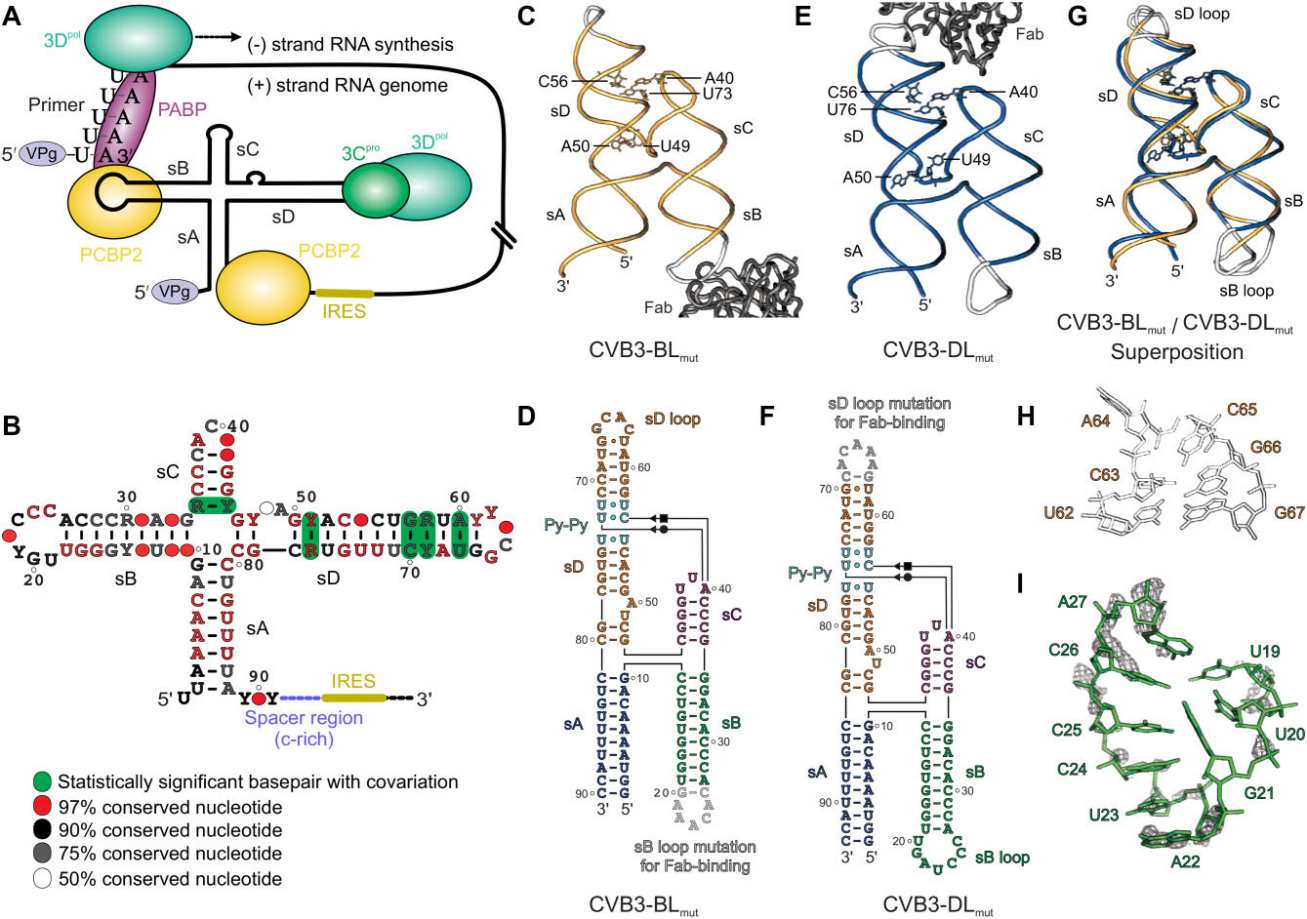
RNA-mediated enteroviral replication and crystal structures of the CVB3 REPLR. (**A**) Schematics showing the process of (–)-strand RNA synthesis during enteroviral genome replication. (**B**) A cloverleaf-like consensus secondary structure of the enteroviral REPLR obtained by aligning over 5000 genome sequences available in the NCBI database. (**C**) The crystal structure of the CVB3-BL_mut_ REPLR solved previously (36) at 1.9 Å resolution and (**D**) its crystal-derived secondary structure (see Supplementary Figure S4 for complete Fab−RNA complex). (**E**) The crystal structure of the CVB3-DL_mut_ REPLR solved at 1.54 Å resolution and (**F**) its crystal-derived secondary structure (see Supplementary Figure S5 for complete Fab–RNA complex). (**G**) The superposition of the common core of CVB3-DL_mut_ (blue) and CVB3-BL_mut_ (yellow) crystal structures (all atoms RMSD = 1.98 Å) without considering the sB and sD loops (gray). (**H**) The structure of the UNCG-type (*N* = any nucleotide) sD loop revealed previously in the CVB3-BL_mut_ construct (36). (**I**) the structure of the sB loop as observed in the CVB3-DL_mut_ construct. The dark gray mesh represents the *2|F*_o_*|– |F*_c_*|* electron density map at 1σ contour level and carve radius 1.8 Å. Notably, a poor electron density map and high crystallographic *B*-factor in the sB loop region suggest its highly dynamic nature (see Supplementary Figure S5).

Previously, the phylogenetic and biochemical probing analyses have proposed that the domain I (called replication-linked RNA, REPLR hereafter) folds into a cloverleaf-like RNA secondary structure with a base-stem, sA, and three stem-loops, sB, sC and sD (Figure 1A). During the (–)-strand RNA synthesis, the first step of viral genome replication, the host poly-C binding protein 2 (PCBP2) interacts with the sB loop and the spacer region between the cloverleaf-like RNA and IRES domain II (16–21). The viral 3CD protein (precursor of the viral protease 3C and RNA-dependent RNA polymerase D) binds to the sD loop through its 3C protease domain (17,22,23). The binding of the host poly(A)-binding protein (PABP) at the 3′ UTR and subsequent interactions between the PABP and the PCBP2-RNA complex promotes the genome circularization (16–20,24–27). The assembly of this ternary RNP complex then recruits the uridylated viral protein VPg and 3CD self-cleaves to yield an active 3D polymerase (28–31), which initiates the (–)-strand RNA synthesis using the viral genome as a template and the uridylated VPg as a primer (Figure 1A). Beyond its role in genome replication, several studies have indicated that the enteroviral REPLRs also serve as a molecular switch to modulate the genome translation and replication through interactions with the host PCBP2 and viral 3CD, where the PCBP2 protein plays a dual role (19,20,27,32–34). The high-affinity binding of the PCBP2 with IRES domain IV is essential for genome translation, whereas its interactions with the REPLR dictate genome replication. While interactions between the PCBP2 and 3CD nor the 3CD and IRES domain IV have been observed, the presence of 3CD has been shown to increase the PCBP2 affinity to the REPLR by about 100-fold, triggering the dissociation of PCBP2 from the domain IV (17,35). However, the structural and mechanistic underpinnings of this genome translation and replication switching and how the REPLRs facilitate this process have not been understood well.

We recently reported a 1.9 Å resolution crystal structure of the REPLR from coxsackievirus B3 (CVB3), a typical enterovirus B species (36). The crystal structure revealed that the CVB3 REPLR adopts an antiparallel H-type four-way junction (4WJ), where the sA-sD and sB-sC helices are co-axially stacked. Interestingly, the structure also revealed a long-range interaction between an adenine (A40) in the sC loop and the non-canonical pyrimidine–pyrimidine (Py–Py) region within the sD stem through an A•C•U base-triple formation, which organized a near-parallel orientation of the sA–sB and sC–sD helices. These structural features were confirmed later in an independent study by Gottipati *et al.* (34) using the CVB3 and poliovirus 1 (PV1) REPLRs. Surprisingly, our isothermal titration calorimetry (ITC) measurements revealed that the sC–sD long-range interactions had little or no influence on the 3C binding (36). However, our preliminary results suggested that the base-triple disruption mutations strongly affect the PCBP2 binding, leading to a hypothesis that the sC–sD interaction stabilizes the H-shaped structure to separate the 3CD and PCBP2 binding sites. This H-shaped configuration positions the two PCBP2 binding sites, the sB loop and the spacer region, side-by-side, suggesting a synergistic interaction of a single PCBP2 with both the sB loop and the spacer region. Nevertheless, how a full-length PCBP2 binds the intact enteroviral REPLRs with the spacer regions remains elusive.

The alignment of enteroviral genome sequences and computation of the consensus secondary structure shows a high degree of conservation among the enteroviral REPLRs (36), including the A•C•U base-triple-forming nucleotides (Figure 1B). Such a high sequence conservation against strong selection pressure suggests that these features are vital for viral genome replication and are likely to be conserved among the enteroviral REPLRs. However, it remains to be demonstrated due to the lack of three-dimensional structures of REPLRs from different enteroviral genotypes. Here, we crystallized and determined four additional structures from the CVB3, rhinovirus B14 (RVB14), and rhinovirus C15 (RVC15) REPLRs using a Fab-assisted RNA crystallography approach. Despite slight variations in the primary sequences and secondary structures, their crystal structures showed the same H-shaped topology and the sC and sD tertiary interaction through the A•C•U base-triple. The conserved structural features observed in these crystal structures also allowed us to compute the structures of enterovirus 71 (EV71), PV1, rhinovirus A2 (RVA2), and enterovirus D68 (EVD68) REPLRs using the FARFAR2 homology modeling (37), which generated models structurally very similar to the crystal structures, demonstrating that the H-shaped topology and sC–sD tertiary interactions are the most conserved structural features across the enteroviral REPLRs.

Further binding studies with intact human PCBP2 with wild-type and mutant RNA constructs in the context of the intact REPLR and spacer region supported the idea that positioning the sB loop and spacer region adjacent to each other facilitates a cooperative binding of a single PCBP2. We revealed that the spacer region represents the high-affinity binding site compared to the sB loop, and any disruption to the integrity of the H-shaped REPLR reduced the affinity of PCBP2 binding due to steric hindrance. While there was no evidence of direct interaction of PCBP2 with the sC–sD tertiary motif, the disruption of the sC–sD interaction through a single mutation diminished the PCBP2 binding even with the sB loop and the spacer region being intact, further supporting that the relative positioning of the sB loop and the spacer is also critical for the effective, high-affinity binding of the PCBP2. In this study, we have also proposed a new model for REPLR-mediated enteroviral replication and transition switching. Although it warrants further investigation, this model mostly agrees with our structural and PCBP2 binding results and the previous functional studies, including the structural basis for 3CD and PCBP2 binding in the clinically isolated enteroviral REPLRs, which contain terminal deletions.

Although the structures and mechanisms of enteroviral REPLRs formed within the 3′ termini of the (–)-strands are mainly unknown, our study provides a structural basis for the (–)-strand synthesis in the wild-type enteroviral genomes as well as in the terminally deleted variants observed in clinical isolates, tissue, and cell cultures. Given the high conservation of REPLRs and the nature of REPLR interactions with proteins, our structural studies may provide opportunities to develop universal drugs that target these viral RNP structures. As a proof-of-concept, we have shown that the presence of oligos complementary to the spacer region within the enteroviral REPLRs could abrogate the PCBP2 binding, which may inhibit enteroviral genome replication. Whereas several aspects of this essential virological phenomenon need to be studied further, our studies here illuminate an initial foundation for pursuing the structure-guided functional and mechanistic studies of enteroviral replication *in vivo*.

## Materials and methods

### Materials

The sequences of all RNA constructs and human PCBP2 used in this study have been provided in Supplementary Table S1. The ASOs were purchased from Integrated DNA Technologies.

### RNA synthesis and purification

RNA constructs for crystallization and PCBP2 binding studies were synthesized by *in vitro* transcription. DNA template with a T7 promoter sequence for transcription reaction was produced by PCR amplification of ssDNA purchased from Integrated DNA Technologies. The first two nucleotides of reverse primer were 2′-OMe modified to reduce the 3′-end heterogeneity of the transcript (38). RNA was transcribed for 3 h at 37°C in a buffer containing 40 mM Tris–HCl, pH 8.0, 2 mM Spermidine, 10 mM NaCl, 0.1 mM EDTA, 25 mM MgCl_2_, 10 mM DTT, 40 U/ml RNase inhibitor, 5 U/ml TIPPase, 5 mM of each NTP, 50 pmol/ml DNA template, and 50 μg/ml homemade T7 RNA polymerase (39). The transcription reaction was quenched by adding 10 U/ml DNase I (Promega) and incubating at 37°C for 30 min. All RNA samples were purified by denaturing polyacrylamide gel electrophoresis (dPAGE). The band was visualized by UV shadowing, excised from the gel, and eluted overnight at 4°C in 10 mM Tris, pH 8.0, 1 mM EDTA, and 300 mM NaCl. The eluted RNA buffer was exchanged with pure water three times using a 10 kDa cut-off Amicon column (Millipore Sigma). RNA was collected, aliquoted into 250 μl fractions, and stored at –80°C until further use.

### Fab expression and purification

Fab BL3–6 expression plasmid was a kind gift from Joseph Piccirilli, the University of Chicago. The Fab was purified according to the published protocols (40–42). Briefly, the plasmid was transformed into 55244 *E. coli* competent cells and streaked into an LB-agar plate with 100 μg/ml of Ampicillin. Several colonies (8-10) were selected to inoculate a 15 ml starter culture and grown at 30°C for 8 h. The starter culture was then used to inoculate 1 liter of 2× YT media, and cells were grown for 24 h at 30°C. For Fab overexpression, the cells were centrifuged at 25°C and 6000 × g for 10 min, resuspended in 1-l phosphate-depleted media and grown for 24 h at 30°C. The cells were harvested by centrifugation at 4°C and 6000 × g for 10 min, resuspended in 1 × PBS, pH 7.4 buffer with 0.01 mg/ml bovine pancreas DNase I (Sigma-Aldrich), and 0.4 mM Phenylmethylsulfonyl fluoride (PMSF), and lysed by sonication (Qsonica, Cole-Parmer). The mixture was first centrifuged at 40 000 × g, the clear lysate was filtered through a 0.45-micron filter (VWR), and the Fab was purified using the Bio-Rad NGC fast protein liquid chromatography (FPLC) system. First, the lysate was passed through a Hi-trap protein A column (Cytiva), and the captured Fab was eluted with 0.1 M acetic acid. The fractions were collected, diluted 10 × using 1 × PBS, pH 7.4 buffer, and loaded into a Hi-trap protein G column (Cytiva). The eluted Fab fractions from the protein G column in 0.1 M glycine, pH 2.7, were collected, diluted 10 × with a 50 mM NaOAc, 50 mM NaCl, pH 5.5 buffer, and loaded into a Hi-trap heparin column (Cytiva). Finally, the Fab fractions eluted from the heparin column by the gradient elution using 50 mM NaOAc, 2 M NaCl, pH 5.5 buffer were collected, and buffer exchanged 3× with 1× PBS pH 7.4 using 30 kDa cut-off Amicon column (Millipore Sigma). The concentrated Fab was collected, analyzed by an SDS-PAGE, and tested for RNase activity using the RNaseAlert kit (Ambion, www.thermofisher.com). Aliquots (∼200 μl) of purified Fab were stored at –80°C.

### Native gel electrophoresis

RNA in water (∼300 nM) was refolded in a buffer containing 50 mM Tris–HCl, pH 7.4, 10 mM MgCl_2_, 100 mM NaCl and 0.1 mM EDTA. RNA was heated at 90°C for 1 min, and an appropriate volume of 10× refolding buffer was added, followed by incubation at 50°C for 10 min and then in ice for 5 min. The refolded RNA was then incubated for 30 min at room temperature with different molar equivalents of the Fab or PCBP2. The protein-RNA complex samples were mixed with an appropriate volume of 6 × native gel loading solution containing 30% glycerol, 0.1% each bromophenol blue, and xylene cyanol. These samples were loaded onto 6% native polyacrylamide gels for PCBP2 and ASOs) or 10% gels for Fab binding and run at 120 V in 0.5× TBE buffer (50 mM Tris–base, 50 mM boric acid, and 1 mM EDTA, pH 7.5) at 4°C. The gels were stained with ethidium bromide and imaged using the Azure 200 gel documentation system (Azure Biosystems).

### Crystallization

The RNA sample was refolded in a folding buffer containing 10 mM Tris–HCl, pH 7.4, 10 mM MgCl_2_, 100 mM NaCl and 0.1 mM EDTA. First, RNA in water was heated at 90°C for 1 min, and an appropriate volume of 10x refolding buffer was added, followed by incubation at 50°C for 10 min and in ice for 5 min. The refolded RNA was then incubated for 30 min at RT with 1.1 equivalent of the Fab and concentrated to 6 mg/ml using a 10 kDa cut-off, Amicon Ultra-1 column (Millipore Sigma). Then, Fab−RNA complexes were passed through 0.2 μm cut-off Millipore centrifugal filter units (www.emdmillipore.com). The Xtal3 Mosquito liquid handling robot (TTP Labtech, ttplabtech.com) was used to set up sitting-drop vapor-diffusion crystallization screens at RT using commercially available screening kits from Hampton Research. All initial crystallization trials were performed by screening 576 conditions for each Fab−RNA complex. The crystals formed within a week in multiple conditions. Select conditions were optimized for pH, precipitant, and salt concentration to grow larger crystals using the hanging drop vapor diffusion method. The crystals grew to full size within a week. Drops containing suitable crystals were brought up to 30% glycerol for cryoprotection without changing the other compositions. Crystals were immediately flash-frozen in liquid nitrogen after being fished from the drops and taken to Argonne National Laboratory to collect X-ray diffraction data. The Fab-CVB3-DL_mut_ yielded crystals in multiple conditions with the best diffracting crystal (1.54 Å resolution) in 0.2 M ammonium phosphate dibasic, 20% polyethylene glycol 3350, pH 8.0, which is different from the Fab–CVB3–BL_mut_ complex crystallization condition (0.01 M magnesium acetate tetrahydrate, 0.05 M MES monohydrate, pH 5.0, 2.5 M ammonium sulfate) reported previously (36).

### Structural data collection, processing and analysis

The X-ray diffraction data sets were collected at the Advanced Photon Source NE-CAT section beamlines 24-ID-C and 24-ID-E. All the datasets were integrated and scaled using RAPD automated programs (https://rapd.nec.aps.anl.gov/rapd/) on-site Initial phases were obtained by molecular replacement with the previously reported structure of Fab BL3–6 (PDB: 8DP3) (36) as the search model using Phaser on Phenix (43). Iterative model building and refinement were performed using COOT (44) and the Phenix package (43). RNA was built unambiguously by modeling the individual nucleotides into the electron density map obtained from the molecular replacement. The refinement used default NCS options and auto-selected TLS parameters in Phenix. Most water molecules were automatically determined by Phenix software during refinements. However, some water molecules were added manually for the positive electron density in the map based on their possibility of forming hydrogen bonds with protein or RNA residues. Solvent-accessible surface area and area of interaction were calculated using (http://www.ebi.ac.uk/pdbe/pisa/) (45). Structure-related figures were made in PyMOL (The PyMOL Molecular Graphics System, Version 2.0 Schrödinger, LLC) or UCSF ChimeraX (46), and figure labels were edited in CorelDraw (Corel Corporation, http://www.corel.com).

### Homology modeling

*De novo* and homology modeling were performed using FARFAR2 on the public Rosetta Commons server ROSIE (https://rosie.rosettacommons.org) (37,47), following the general methodology described in Watkins *et al.* (37). The template was created by extracting the A•C•U base-triple-forming nucleotides from the experimentally derived CVB3 REPLR structural model. To make the 4WJ homology template, two base pairs from each sA, sB, sC and sD helices flanking the 4WJ in the CVB3 crystal structure were taken. To determine the average RMSDs, the predicted homology models with the top five lowest energy were superimposed with crystal structures in PyMOL or UCSF ChimeraX (46).

### Human PCBP2 expression and purification

The full-length human PCBP2 (residues 11–359) with C-terminal 6x-His tag was expressed and purified using previously described protocols with some modifications (16,35). Briefly, the codon-optimized DNA sequence encoding the human PCBP2 protein was cloned into the pET-22b(+) vector between NdeI and XhoI restriction sites (GenScript, https://www.genscript.com). The lysis buffer contained 50 mM Tris (pH 7.5), 300 mM NaCl, and 20 mM imidazole, whereas the HisTrap column elution buffer contained 50 mM Tris (pH 7.5), 300 mM NaCl and 250 mM imidazole. The eluted fractions were collected, dialyzed against a buffer containing 50 mM Tris (pH 7.5), 100 mM KCl, 1 mM EDTA and 5% glycerol, and purified further by size-exclusion chromatography with HiLoad® 26/600 Superdex® 75 pg column. The single-peak protein fractions were pooled and concentrated using the Amicon centrifugal filters (molecular weight cut-off 30 kDa), flash-frozen with liquid N_2_ in small aliquots and stored at –80°C until further use.

### Biolayer interferometry (BLI)

The experiments were performed in the Octet R2 system (Sartorius) using the Octet® anti-penta-HIS (HIS1K) biosensors (Sartorius). The His-tagged PCBP2 protein (15 μg/ml concentration) was immobilized on the biosensor surface after hydrating the biosensor for 10 min in the assay buffer (PBS pH 7.4). These protein-immobilized biosensors were then dipped into varying concentrations of the RNA solution and a reference sample (assay buffer) for the background correction. The BLI responses as a function of time data were recorded and then processed by aligning the baselines using the BLI system-integrated software. To determine the *K*_d_ from the BLI data, the responses from the plateau region of the association curves (i.e. average responses from the 110–115 s region for each concentration were plotted against the RNA concentration and fitted using the following binding isotherm.

$Response\ ( Y ) = \ \frac{{{R}_{max\ }[ {RNA} ]}}{{({K}_d\ + \ [ {RNA} ]}}$ …… (4), where *R*_max_ refers to the maximum response due to the RNA binding to the protein. A typical dataset and *K*_d_ determination procedure is shown in Supplementary Figure S2.

## Results

### CVB3 REPLR structure shows no long-range interactions between the sB and sD loops

We previously crystallized and determined the 1.9 Å resolution structure of the CVB3 REPLR in complex with Fab–BL3–6 as a crystallization chaperone, where the Fab-binding sequence replaced the sB loop of the REPLR (Figure 1C, D) (36). To access the potential influence of the Fab binding tag sequence and subsequent Fab binding on the H-shaped conformation, we crystallized and determined the structure of the same CVB3 REPLR using the same Fab-assisted crystallography approach, but this time, the Fab-binding tag replaces the sD loop rather than the sB loop. Both sD and sB loop mutant constructs for crystallization (designated CVB3-DL_mut_ and CVB3-BL_mut_, respectively) bound the Fab BL3–6 similarly (Supplementary Figure S3), indicating that sD and sB loops are equally accessible to the Fab, consistent with the H-shaped topology and antiparallel positioning of the sB and sD loops as observed in our previous CVB3–BL_mut_ crystal structure (36). Moreover, our previous 3C binding studies with the intact CVB3 REPLR and isolated sD stem-loop constructs (36) and the human PCBP2 binding results with the CVB3 and RVB14 REPLR constructs, as discussed below, also support no apparent long-range tertiary interactions between the sB and sD loops.

Following the binding studies, we advanced the CVB3-DL_mut_ construct in complex with Fab BL3–6 to crystallization and structure determination (see Materials and methods). The Fab−RNA complex structure was solved at 1.54 Å resolution (Figure 1E, F) using Fab BL3–6 (PDB: 8DP3) (36) as a molecular replacement model for the initial phasing. Details of the data collection and refinement statistics are shown in Supplementary Table S2, and the final structural model with the *2|F*_o_*| – |F*_c_*|* electron density map and colored according to the crystallographic *B*-factors are shown in Supplementary Figure S4. Despite different crystallization conditions, the crystallographic asymmetric unit (*a* = 145.99 Å, *b* = 80.37 Å, *c* = 87.34 Å, α = 90.00°, β = 111.17° and γ = 90.00°) contained a single Fab-CVB3-DL_mut_ complex packed in the *C*12_1_ space group, almost identical to that for the Fab-CVB3–BL_mut_ complex (Figure 1C, D). Expectedly, the common core (without the sB or sD loops) of the CVB3–BL_mut_ and CVB3–DL_mut_ structures (Figure 1G), including the sC–Py–Py tertiary interactions, were superimposable (all atoms RMSD = 1.98 Å, see Supplementary Figure S5 for overall alignment of Fab−RNA complexes), suggesting that grafting of the Fab binding tags does not influence the H-shaped architecture of the CVB3 REPLR. Similarly, the recently published structure of the same REPLR that was crystallized using tRNA scaffold incorporated in the sA helix (34) also superimposes with our crystal structures (RMSD = 1.62 Å and 1.48 Å with the common cores of the CVB3–BL_mut_ and CVB3–DL_mut_ structures), confirming that CVB3 REPLR is a modular domain preorganized into H-shaped architecture that positions the sB and sD loops (PCBP2 and 3C binding sites) distal to each other. Such positioning perhaps prohibits the tertiary interactions between these two protein binding sites necessary for the (–)-strand RNA synthesis during viral genome replication. Whereas we showed that the sD-loop in the CVB3-BL_mut_ construct adopts a UNCG (N = A, U, G and C) type tetraloop (Figure 1H), the modeling of the sB-loop in the CVB3-DL_mut_ construct was more ambiguous due to a poor electron density map observed in this region (Figure 1I). Also, high crystallographic *B*-factors in this region suggest the highly dynamic nature of the B-loop (see Supplementary Figure S5), which might be stabilized upon PCBP2 binding.

### Crystal structures of RV REPLRs revealed topologies identical to those of CVB3 and PV1

The *enterovirus* genus comprises several human viruses, including the four enteroviruses (A, B, C and D) and three rhinoviruses (A, B and C) species (48–51). Comparisons of our two CVB3 (enterovirus B) and previously reported PV1 (enterovirus C) REPLR crystal structures (34,36) suggest that the H-shaped topology and the sC–sD long-range interactions observed in REPLR structures of these two species are very similar. To further demonstrate that such topological conservation exists across the enteroviral REPLRs, we sought to determine the high-resolution crystal structures of the five additional REPLRs, each representing a genotype from the enterovirus A and D (EVA71 and EVD68) and the rhinovirus A to C (RVA2, RVB14 and RVC15) species. Because Fab-assisted crystallography has effectively determined the structure of our CVB3 constructs, we pursued the same approach with Fab BL3–6 as a crystallization chaperone. For this, we prepared two crystallization constructs for each REPLR by replacing the wild-type sB or sD loop with a Fab BL3–6 binding tag, 5′ GAAACAC 3′, which enabled Fab−RNA complex formation for each crystallization construct (Supplementary Figures S6 and S7) and allowed subsequent crystallization of the complex.

Following the binding studies with Fab BL3–6, we set up the crystallization trials with both sB and sD loop mutant constructs (designated as ‘BL_mut_’ and ‘DL_mut_’, respectively) for all five REPLRs, each in complex with Fab BL3–6 (see Supplementary Table S1 for all sequences used in this study). We observed crystal hits for RVA2–BL_mut_, RVA2–DL_mut_, RVB14–DL_mut_, RVC15–BL_mut_, RVC15–DL_mut_, EV71–BL_mut_ and EV71–DL_mut_. However, RVB14–BL_mut_, EV68–BL_mut_ and EV68–DL_mut_ constructs did not produce any crystal hits, unlike the robust crystallization of the analogous CVB3 constructs, although these RNAs have similar sequences and secondary structures. Similar trials for all these constructs without the Fab BL3–6 yielded no crystals, suggesting a significant role of the Fab in the crystal packing. Further optimization of pH, precipitant, salt, and additives yielded crystals for RVB14–DL_mut_, RVC15–BL_mut_ and RVC15–DL_mut_ constructs that diffracted to 2.20, 2.97 and 2.54 Å resolution, respectively. Further analysis of the diffraction data shows the complexes Fab BL3–6–RVB14–DL_mut_ and Fab BL3–6–RVC15–DL_mut_ crystallized in a lattice space group *P*12_1_1 with two Fab−RNA complexes per crystallographic asymmetric unit (*a* = 70.92 Å, *b* = 79.21 Å, *c* = 141.32 Å, α = 90.00°, β = 98.87° and γ = 90.00°, and *a* = 52.99 Å, *b* = 106.37Å, *c* = 142.82 Å, α = 90.00°, β = 99.23° and γ = 90.00°, respectively). In contrast, the Fab BL3–6–RVC15–BL_mut_ was crystallized in a lattice space group *I*2_1_2_1_2_1_ with one Fab−RNA complex per crystallographic asymmetric unit (*a* = 103.22 Å, *b* = 121.85 Å, *c* = 174.66 Å, α = 90.00°, β = 90.00° and γ = 90.00°). After processing X-ray diffraction data, the molecular replacement using a previous crystal structure of Fab BL3–6 (PDB: 8DP3) (36) readily provided the initial phases, and the resulting high-resolution electron density map facilitated unambiguous modeling of the nucleotides to build the final RNA models through iterative rounds of model building and refinement. Supplementary Table S2 shows the data collection and refinement statistics in detail, and all Fab−RNA final structural models with corresponding *2|F*_o_*|–|F*_c_*|* electron density maps and colored according to the crystallographic *B*-factors are shown in Supplementary Figures S8–S10.

**Figure 2.**
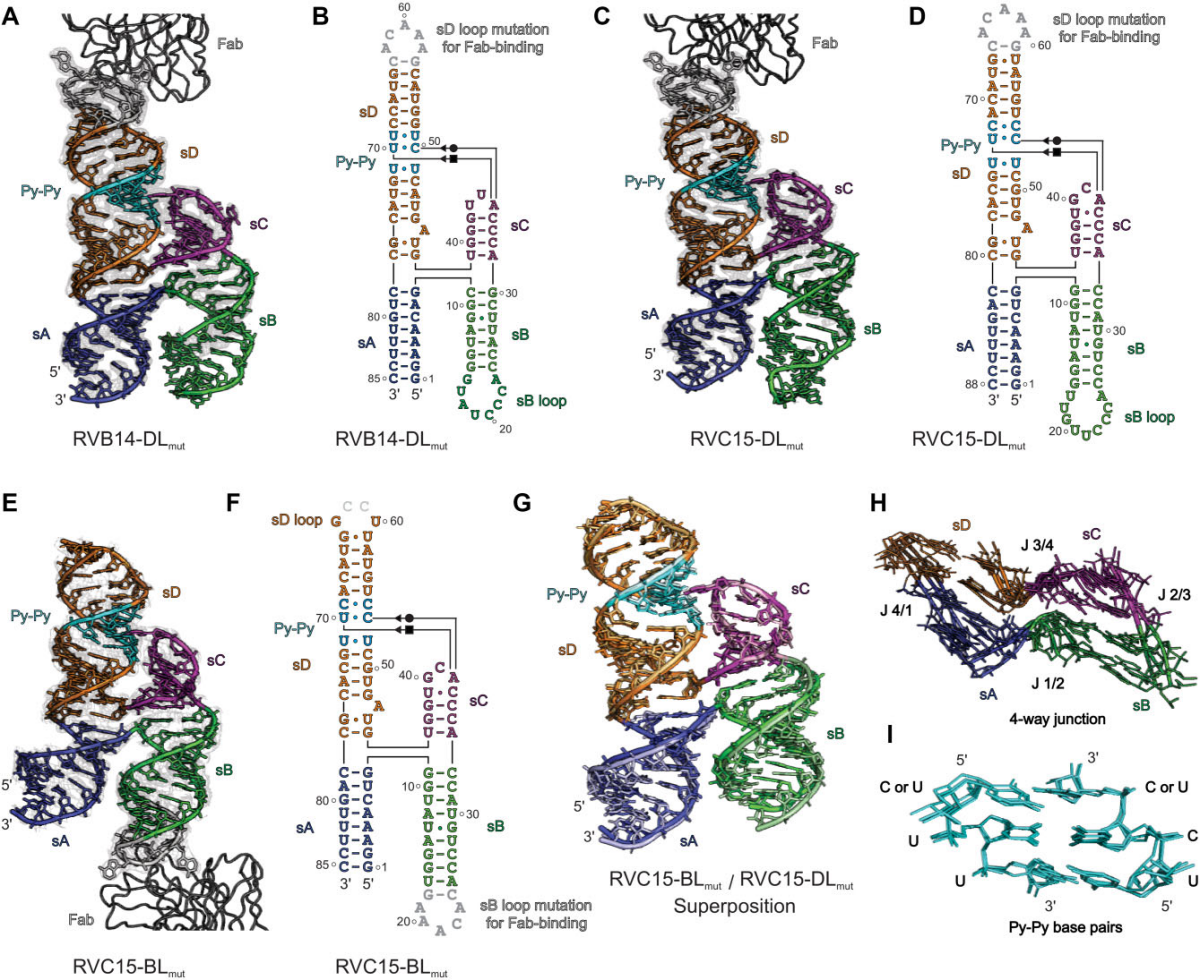
Crystal structures of the RVB14 and RVC15 REPLRs. (**A**) The crystal structure of the RVB14–DL_mut_ REPLR solved at 2.2 Å resolution (see Supplementary Figure S8 for complete Fab−RNA complex) and (**B**) its crystal-derived secondary structure. (**C**) The crystal structure of the RVC15–DL_mut_ REPLR solved at 2.54 Å resolution (see Supplementary Figure S9 for complete Fab−RNA complex) and (**D**) its crystal-derived secondary structure. (**E**) The crystal structure of the RVC15–BL_mut_ REPLR solved at 2.97 Å resolution (see Supplementary Figure S10 for complete Fab−RNA complex) and (**F**) its crystal-derived secondary structure. (**G**) The superposition of the common core of RVC15–BL_mut_ and RVC15–DL_mut_ (pale-colored) crystal structures (all atoms RMSD = 0.972 Å) without considering the sB and sD loops. (**H**) The superposition of the 4WJ region of the CVB3, RVB14 and RVC15 REPLRs. (**I**) The superposition of the Py–Py region within the sD helix of CVB3, RVB14 and RVC15 REPLRs. The gray mesh in A, C and E represent the *2|F*_o_*| – |F*_c_*|* electron density map at 1σ contour level and carve radius 1.8 Å. The panels are colored analogously for facile comparisons.

Despite being crystallized in different conditions and space groups, the crystal structures of RVB14 REPLR (Figure 2A, B) showed remarkable similarity with the CVB3 REPLRs (Figure 1C-F), including the Py–Py base pairs, sD dinucleotide bulge, and the sC–Py–Py tertiary interactions through the A•C•U base-triple formation. Consistent with the CVB3–DL_mut_ (Figure 1E, F) and CVB3–BL_mut_ (Figure 1C, D), the two RVC15 REPLR structures, RVC15–DL_mut_ (Figure 2C, D) and RVC15–BL_mut_ (Figure 2E, F), were very similar (Figure 2G, RMSD = 0.972 Å for the superposition of the common cores without sB and sD loops). Overall, the crystal structures of the REPLRs from CVB3, RVB14, RVC15 and PV1(34) revealed a high degree of topological conservation among the enteroviral REPLRs, including the conserved 4WJ structure (Figure 2H) and Py–Py helix (Figure 2I), where the central C•U is involved in the A•C•U base-triple formation. Notably, in all structures of these REPLRs, the modeling of the sB-loop was ambiguous due to the poor electron density map in the region, which suggests that the sB-loop with the highest crystallographic B-factors is more dynamic compared to the other areas within the enteroviral REPLR structures (Supplementary Figures S5, S8 and S9), indicating that stabilization of the sB-loop may be influenced by PCBP2 binding. Although these viruses cause different diseases, the structural homology of the enteroviral REPLRs indicates their functional similarities in the genome replication mechanisms across enteroviruses, providing tremendous opportunities for developing universal therapeutics that target the enteroviral replication platform.

### Enteroviral REPLRs exhibit subtle variations in local structural features

Although our CVB3, RVB14 and RVC15 REPLRs crystal structures exhibited a highly conserved H-shaped topology with sC–sD tertiary interaction, they revealed some subtle variations in their local structural features. While the 4WJ without unpaired nucleotides within the junction appears conserved in these crystal structures (Figure 2H), the UA dinucleotide bulge structure within the sD helix varied slightly. In the bulge of the CVB3-DL_mut_ construct (Figure 1E, F), the U49 is flipped out, and A50 forms a base triple involving the A50, C48 and G82 through hydrogen bonding interactions, where C48 and G82 form the Watson-Crick base pair, the A50 Sugar-Edge interacts with C48 O2 and G82 N2, and the A50 N1 contact G82 2′OH through a hydrogen bonding (Supplementary Figure S11). This bulge structure was slightly different in the previously reported CVB3–BL_mut_ construct (Figure 1C, D), where U49 remained within the helical stack and was involved in the U49•G51–C78 base-triple formation, and the A50 flipped out of the helix and interacted with the symmetry-mate Fab molecule (36), which is further stabilized by the hydrogen bonding interactions of the G51 O4′ and A50 N3 with the A50 2′OH (Supplementary Figure S11). Nevertheless, these structural variations may reflect a high dynamicity of the bulge, and thus, each crystal structure perhaps represents a sampled conformation of the bulge within these CVB3 REPLR crystal structures. Intriguingly, with the formation of U43•G76 and U46•G76 wobble pairs, the RVB14 and RVC15 REPLR crystal structures showed a single-nucleotide bulge with flipped-out A44 and A47, respectively (Figure 2B, D and F, Supplementary Figure S11). Notably, such an arrangement forms 2 bp between the 4WJ and the bulge in all these structures, suggesting that a single-nucleotide sD bulge is sufficient for the 3C binding, which is consistent with our 3C binding studies with the CVB3 REPLR (36). As the U is less conserved than A within this UA bulge among enteroviral REPLRs (Figure 1B), the bulge structure with flipped-out A might be more relevant for interactions with the 3C protein.

While the helical lengths (number of base pairs) for the sC (four base-paired helices) and sD (14-based-paired helix) were conserved among the enteroviral REPLR structures, we observed subtle structural variations in the sC and sD loops. The mFold (52) predicted secondary structures suggest that whereas RVB14 contains a UAU tri-loop, unlike an sD tetraloop present in all other enteroviral REPLRs, the RVC15 and PV1 were expected to form the sC pentaloops (CACGU), unlike a triloop found in other enteroviral REPLRs (Supplementary Figure S12). Interestingly, with U41 being flipped out in the RVC15 REPLR, our CVB3, RVB14 and RVC15 REPLR crystal structures all essentially contain an sC triloop. Such arrangement not only maintains the four base-paired sC helix in all enteroviral REPLRs but also precisely positions the conserved A within this sC loop (A40, A35 and A38 in the CVB3, RVB14 and RVC15 REPLRs, respectively) to dock into the sD Py–Py helix. It is apparent that the sD bulge and the sC–sD tertiary interactions maintain the structural integrity of the enteroviral REPLRs to offer a pre-organized platform for the 3C binding (36). While the length of the sB helix is longer for CVB3 (9 base pairs) and RVC15 (8 bp) compared to that for RVB14 (7 base pairs), which may have implications in PCBP2 binding (discussed later), the sB loops seem more dynamic with the highest crystallographic *B*-factors (Supplementary Figures S8 and S9). As the modeling of the sB loop nucleotides was ambiguous due to poor electron density maps even for the 1.54 Å resolution structure of the CVB3 REPLR, we have not further discussed the structural features of these sB loops.

### Conserved structural features allowed homology modeling of all seven enteroviral REPLRs

Sequence alignments of over 5000 genomes for seven different human enterovirus species, including the enteroviruses A, B, C and D and rhinoviruses A, B, and C, show a high degree of sequence conservation among the enteroviral REPLRs (36). Accordingly, the consensus secondary structure (Figure 1B) predicted by the R-Scape (RNA Structural Covariation Above Phylogenetic Expectation) (36) revealed highly conserved features within each subdomain of these REPLRs. Notably, the absolute conservation of the adenosine within the sC-loop (for example, A40 in the CVB3-BL_mut_ construct) and the Py–Py helix within the sD stem region is consistent with their structural roles in stabilizing the 4WJ structure of the REPLRs as observed in our CVB3, RVB14 and RVC15 crystal structures. Such structural features remain preserved across enteroviral REPLRs regardless of the modifications (mutations or insertions) made in the sB and sD loops or sA helix, as illustrated by the CVB3, RVB14, RVC15 and PV1 (34) crystal structures. However, we failed to obtain the high-resolution structures for EV71, PV1, EVD68 and RVA2 REPLRs using the Fab-assisted crystallography approach because of no or poorly diffracting Fab−RNA complex crystals. While we continue our efforts to determine the high-resolution structures of these RNAs, the observed high conservation of the sequence, secondary structures, and tertiary interactions among the crystal structures of CVB3, RVB14 and RVC15 REPLRs led us to examine whether these interactions help predict the structures of other enteroviral REPLRs *in silico*.

**Figure 3.**
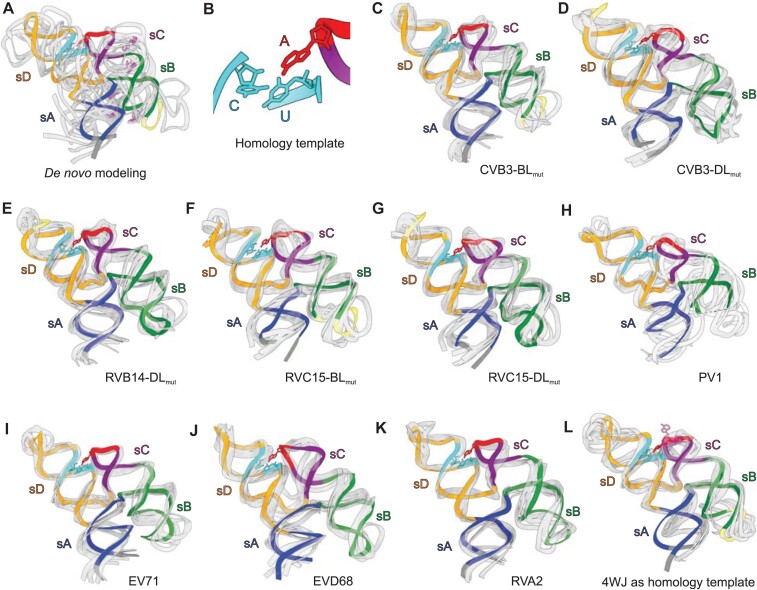
Homology modeling of the REPLRs from seven enteroviral species. (**A**) The five lowest-energy models of the CVB3 REPLR predicted *de novo* superimposed with the CVB3-BL_mut_ crystal structure. (**B**) The A•C•U base-triple use in the homology modeling of REPLRs from seven different enteroviral species (i.e. enterovirus A–D and rhinovirus A–C). (**C**) the five lowest-energy homology models of CVB3 REPLR superimposed with the CVB3-BL_mut_ and (**D**) CVB3–DL_mut_ crystal structure (RMSD = 4.4 ± 0.7 and 4.5 ± 0.9 Å, respectively). (**E**) The five lowest-energy homology models of the RVB14 REPLR superimposed with the RVB14-DL_mut_ crystal structure (RMSD = 4.6 ± 0.8 Å). (**F**) The five lowest-energy homology models of the RVC15 REPLR superimposed with the RVC15–BL_mut_ and (**G**) RVC15–DL_mut_ crystal structure (RMSD = 3.4 ± 0.4 and 4.1 ± 0.7 Å, respectively). (**H**) The five lowest-energy homology models of the PV1 REPLR superimposed with its previously reported crystal structure RMSD = 6.2 ± 1.6 Å). (**I**) The five lowest-energy homology models of EV71, (**J**) EVD68 and (**K**) RVA2 REPLRs with the lowest-energy model colored analogously as the CVB3 REPLR crystal structures. (**L**) The five lowest-energy homology models for the CVB3 REPLR computed using 4WJ as a homology template superimposed with the CVB3-BL_mut_ crystal structure. All predicted models are colored gray, and the crystal structures (in panels A, C–H and L) or the lowest-energy model (in panels I–K) are colored analogously as the CVB3 REPLR crystal structures. The Fab-binding loop has been colored yellow for the crystal structures in panels A, C–G and L. The A40s in each predicted model in panels A and L are colored light purple to indicate their location within the corresponding structure. Supplementary Figure S13 shows the crystal and model-derived secondary structures for each representative REPLR.

Based on the homologous sequences and predicted secondary structures of the enteroviral REPLRs compared to crystal-derived CVB3 structure, we computed the three-dimensional models for all seven enteroviral REPLRs *in silico* using the FARFAR Rosetta homology modeling protocol (37). First, to gauge the effectiveness of this approach, we compared the results obtained for the CVB3 REPLR from a blind *de novo* FARFAR modeling with its homology modeling using the A40–Py–Py interaction as a seeding template. This blind *de novo* modeling resulted in models inconsistent with the observed crystal structure, including the orientation of the 4WJ reversed in some instances (Figure 3A). However, the homology modeling using the A40–Py–Py as a seeding template (Figure 3B) yielded almost identical 4WJ orientations in all top five lowest-energy models with an overall average RMSD of 4.5 ± 0.9 Å (Figure 3C, D). Next, we applied this method to compute the structures of the other six enteroviral REPLRs using the A40–Py–Py interaction as a seeding template. The top five lowest-energy models of all six REPLRs appear similar in the overall architecture to the crystal structures of the CVB3 REPLR. Interestingly, the lowest-energy models for RVB14 and RVC15 superimpose well with their corresponding crystal structures (Figure 3E–G). Additionally, the five lowest-energy homology models computed for PV1 REPLR are also very similar (Figure 3H) to its recently reported crystal structure (34). Although high-resolution structures are not available, the five lowest-energy homology models of the EV71, EVD68 and RVA2 REPLRs appear very similar to the CVB3 REPLR crystal structure (Figure 3I-K), suggesting that the H-shaped topology along with long-range interactions between the sC-loop and Py–Py helix is perhaps the most common structural feature among the enteroviral REPLRs (see Supplementary Figure S13 for the model-derived secondary structures of all seven enteroviral REPLRs). Furthermore, using an intact 4WJ as a seeding template (two base pairs of each sA, sB, sC and sD helices flanking the junction) yielded the homology models inconsistent with the observed crystal structures for the CVB3, RVB14, RVC15 and PV1 REPLRs (Figure 3L), implying that the sC–Py–Py tertiary interaction is the crucial anchoring point that stabilizes the REPLR’s 4WJ architecture for positioning the 3C and PCBP2 binding sites antiparallel to each other.

### 3′ spacer is the high-affinity binding site for human PCBP2 compared to the sB loop

**Figure 4.**
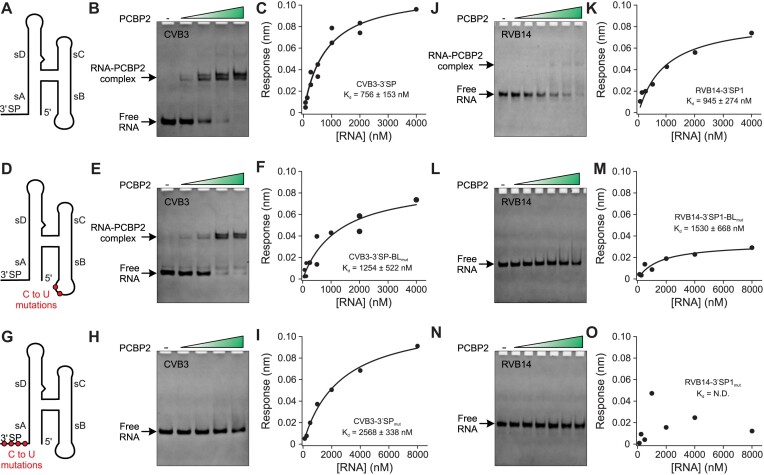
Binding interactions of full-length human PCBP2 with enteroviral REPLRs. (**A**) Schematics of the REPLR construct for the binding studies with an H-shaped core and the C-rich 3′SP sequence. (**B**) A native gel showing the RNA–PCBP2 complex with formation with CVB3 3′SP construct (300 nM). (**C**) A BLI-based binding isotherm showing apparent binding affinity (*K_d_*) measurements for the CVB3 3′SP-PCBP2 interactions. (**D**) The CVB3 3′SP sB-loop mutant construct (CVB3–3′SP–BL_mut_) with C25U and C26U mutations, (**E**) in native gel showing the PCBP2 binding with the CVB3–3′SP–BL_mut_ (300 nM) and (**F**) a BLI-based binding isotherm showing its apparent binding affinity. (**G**) The CVB3-3′SPmut construct with C95U, C97U, C101U and C103U mutations. (**H**) A native gel showing the PCBP2 binding with the CVB3-3′SPmut (300 nM), and (**I**) a BLI-based binding isotherm showing its apparent binding affinity. (**J**) A native gel for binding of RVB14-3′SP1 construct (300 nM) and (**K**) a BLI-based binding isotherm showing its apparent binding affinity measurements. (**L**) A native gel and (**M**) the BLI-based binding isotherm for the interactions of the PCBP2 with the RVB14–3′SP1 sB-loop mutant (i.e. RVB14–3′SP1–BLmut containing C21U and C22U mutations). (**N**) a native gel and (**O**) the BLI-based binding isotherm for the interactions of the PCBP2 with the RVB14-3′SP1mut construct containing C83U, C85U, C89U and C92U mutations in the 3′SP region. The green color-gradient triangle shows the PCBP2 concentration range from 0.3–1.2 μM (0.3 μM increments in each lane) and 0.3–3.1 μM (0.3, 0.6, 1.2, 1.8, 2.5 and 3.1 μM in each lane from left to right) for the CVB3 and RVB14 constructs, respectively. The filled circles in binding isotherms represent the BLI responses for a given RNA concentration, and the solid curve depicts the fitting (see methods for details). N.D. depicts not determined, and the reported *K*_d_ values are the mean ± standard deviation (*n* = 3).

Previous biochemical studies showed that PCBP2 recognizes two sites near the enteroviral replication platform – the sB loop and spacer sequence between the REPLR’s 3′ end and the IRES domain II. Our structural studies with seven enteroviral species revealed that the conserved H-shaped REPLR structure indeed positions the sB loop and 3′ spacer (3′SP) in proximity, perhaps facilitating a cooperative binding of the PCBP2. To understand the molecular and structural basis of such interactions, we recombinantly purified the full-length human PCBP2 and performed binding studies with REPLR constructs for CVB3 and RVB14 using native gel electrophoresis and bio-layer interferometry (BLI) measurements (see methods for details). First, we found that the CVB3 REPLR construct with the 3′SP sequence (CVB3-3′SP, Figure 4A) binds the PCBP2 with high affinity (*K*_d_ = 756 ± 153 nM, Figure 4B, C). Interestingly, while the cytosine mutations (C25U and C26U) in the sB-loop in the context of the CVB3-3′SP construct (Figure 4D, CVB3-3′SP-BL_mut_) slightly reduced the binding affinity to the PCBP2 (*K*_d_ = 1254 ± 522 nM, Figure 4E, F), similar mutations of the cytosines (C95U, C97U, C101U and C103U) to the 3′SP (Figure 4G, CVB3-3′SP_mut_) significantly reduced the PCBP2 binding (*K*_d_ = 2568 ± 338 nM, Figure 4H, I), suggesting that the 3′SP sequence constitute a higher affinity binding site for PCBP2 compared to the sB loop. Furthermore, a construct with these mutations in both the sB loop and 3′SP (CVB3–3′SP_mut_–BL_mut_) showed no detectable binding with the PCBP2 (Supplementary Figure S14), supporting that the sB loop and the 3′SP are likely the two major interaction sites for the PCBP2 within the enteroviral platform. However, these experiments do not rule out the allosteric effect of such mutations on PCBP2 binding affinity. Additionally, extending the 3′SP by two more adenines (CVB3-3′SP_AA_, *K*_d_ = 585 ± 111 nM) or a portion of the IRES domain II stem–loop (CVB3–3′SP_HP_, *K*_d_ = 600 ± 124 nM) had no significant influence on the PCBP2 binding compared to the CVB3–3′SP, suggesting that 11-nt poly-C 3′SP sequence (5′ CCCCCUCCCCC) is required but sufficient for the high-affinity PCBP2 binding (Supplementary Figure S14). Notably, the PCBP2 binding with the wild-type CVB3–3′SP and its mutant with Fab BL3–6 binding tag as the sD-loop CVB3–3′SP-DL_BL3–6_ were very similar, suggesting that the grafting of the Fab-binding sequence did not influence the overall folding of the REPLR (Supplementary Figure S15). Moreover, the strong binding of PCBP2 with the CVB3–3′SP–DL_BL3–6_ in the presence of Fab BL3–6 also supports the distal location of the sD and sB loops within the REPLR with no tertiary contacts between them (Supplementary Figure S15). Furthermore, a CVB3–3′SP2 construct without the first two G–C pairs that stabilized the sA stem in our crystallization constructs showed similar binding affinity *(K*_d_ = 513 ± 154 nM) as the CVB3-3′SP (*K*_d_ = 756 ± 153 nM), indicating that such stabilization has no significant impact on the REPLR structure and PCBP2 binding (see Supplementary Figure S15).

To explore whether the PCBP2 interactions are analogous in other enteroviral REPLRs, we also performed binding studies with the RVB14 REPLRs. Similar to our observations for the CVB3–3′SP constructs, an RVB14 3′SP construct (RVB14–3′SP1) bound the PCBP2 with *K*_d_ = 945 ± 274 nM (Figure 4J, K). Consistently, whereas the C21U and C22U mutations in the sB loop (RVB14–3′SP1–BL_mut_) preserved a high-affinity binding (*K*_d_ = 1530 ± 688 nM) with the PCBP2 (Figure 4L, M), the cytosine mutations (C83U, C85U, C89U and C92U) to the 3′SP (RVB14–3′SP1_mut_) abolished the binding (Figure 4N, O), suggesting that the RVB14 3′SP also represents the higher affinity site for the PCBP2 binding compared to the sB loop. Moreover, consistent with the CVB3 constructs, a construct with these mutations in both the sB loop and 3′SP (RVB14–3′SP1_mut_–BL_mut_) showed no detectable binding with the PCBP2, suggesting that the nature of the PCBP2 binding is similar in the CVB3 and RVB14 REPLRs (Supplementary Figure S16). However, unlike with the CVB3 construct, an extension of the 3′SP to include an additional 17-nt cytosine-rich sequence (RVB14–3′SP2) significantly increased the PCBP2 binding affinity compared to the RVB14–3′SP1 (*K*_d_ = 247 ± 74 nM and 945 ± 274 nM, respectively), suggesting that RVB14 needs a much longer 3′SP (17-nt) for achieving the similar high-affinity PCBP2 as the shorter 11-nt CVB3 3′SP (Supplementary Figure S16). Although the H-shaped topology is preserved across enteroviral REPLRs that position the sB loop and 3′SP on the same side, the more extended 3′SP in the RVB14 REPLR perhaps compensates for its shorter sB helix (see Figures 1F and 2D) precisely posing the two PCBP2 binding sites for high-affinity interactions.

### sC–Py–Py tertiary interaction modulates the human PCBP2 binding to enteroviral REPLRs

**Figure 5.**
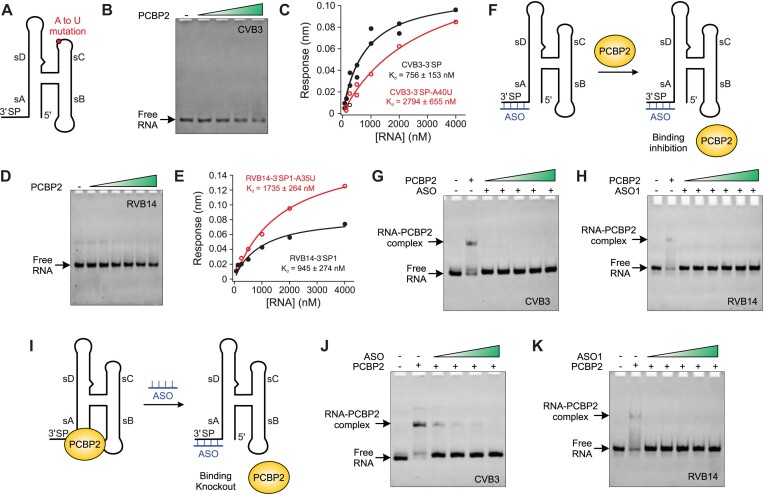
Modulation of human PCBP2 binding to enteroviral REPLRs and its inhibition by the ASOs. (**A**) Schematics CVB3 3′SP construct with A40U mutation that disrupts the A•C•U base-triple. (**B**) A native gel and (**C**) the BLI-based binding isotherm for the interactions of the PCBP2 with the CVB3-3′SP_A40U_ construct. (**D**) A native gel and (**E**) the BLI-based binding isotherm for the interactions of the PCBP2 with the RVB14–3′SP1_A35U_ construct. The data for the corresponding non-mutant constructs (CVB3 3′SP and RVB14-3′SP1) are also shown, and the data points (filled black circles and red open circles for the wild-type and the mutant constructs) and fitting curves (solid black and red curves for the wild-type and mutant constructs) are colored analogously for facile comparison. (**F**) Schematics showing the binding inhibition of PCBP2 with the enteroviral REPLRs by pre-incubated ASO. (**G**) A native gel showing the inhibition of PCBP2 binding with CVB3 3′SP construct (300 nM) in the presence of the ASO (600 nM), and (h) a similar native gel showing the inhibition of PCBP2 binding with RVB14 3′SP1 construct (300 nM) in the presence of the ASO1 (600 nM). The green color-gradient triangle shows the PCBP2 concentration range from 0.3–1.2 μM (0.3 μM increments in each lane) and 0.3–3.1 μM (0.3, 0.6, 1.2, 1.8, 2.5 and 3.1 μM in each lane from left to right) for the CVB3 and RVB14 constructs, respectively. (**I**) Schematics showing the knocking out of the PCBP2 binding with the enteroviral REPLRs by the ASO. (**J**) A native gel showing the displacement of PCBP2 (1.2 μM) from the PCBP2–CVB3–3′SP complex by the ASO in a concentration-dependent manner and (**K**) a similar native gel showing the displacement of PCBP2 (3.1 μM) from the PCBP2–RVB14–3′SP1 complex by the ASO1. The green color-gradient triangle shows the ASO concentration range from 0.6–4.8 μM (0.6, 1.2, 2.4, 4.8 μM in each lane from left to right) and 0.3–4.8 μM (0.3, 0.6, 1.2, 2.4, 4.8 μM in each lane from left to right) for the CVB3 and RVB14 constructs, respectively.

Our previous studies with intact CVB3 REPLR and the isolated sD constructs revealed that tertiary interaction between the sC loop A40 and the sD stem Py–Py helix has almost no effect on the 3C binding to the enteroviral REPLRs (36). Nevertheless, the antiparallel positioning of the 3C and PCBP2 binding sites within the H-shaped crystal structures of the enteroviral REPLRs indicates that such tertiary interaction could be significant for the stability of REPLRs and, thus, interactions with another binding partner, PCBP2. Furthermore, the H-shaped architecture suggests that the proposed PCBP2 binding sites, the sB loop and 3′SP, reside near each other, raising the possibility of RNA-RNA interactions between themselves. To test these hypotheses and investigate how this conserved tertiary interaction modulates the PCBP2 binding to the REPLRs, we performed gel electrophoresis and BLI measurements for human PCBP2 binding with the CVB3 A40U and RVB14 A35U mutant REPLRs containing the 3′SP sequence (Figure 5A). Interestingly, the A40U mutant construct CVB3-3′SP_A40U_ binds the PCBP2 with ∼4 times lower affinity (*K*_d_ = 2798 ± 655 nM) compared to its corresponding non-mutant CVB3-3′SP construct (*K*_d_ = 756 ± 153 nM, Figure 5B, C). These results were also consistent with the binding of PCBP2 to the RVB14-3′SP1 (*K_d_* = 945 ± 274 nM) and RVB14-3′SP1_A35U_ (*K*_d_ = 1735 ± 264 nM) constructs (Figure 5D, E), suggesting that the instability the CVB3–3′SP_A40U_ and RVB14–3′SP1_A35U_ REPLRs caused by the A•C•U base-triple the disruption hinders the cooperative binding of the PCBP2. It is possible that the juxtaposition of the sB and 3′SP enables long-range tertiary interactions between them, which may be required for the high-affinity PCBP2 binding, and thus, the hinge-like motion induced by the A•C•U base-triple disruption may break such sB–3′SP interactions, diminishing the PCBP2 binding. Whereas our current experiments do not entirely rule out such a possibility, little impact on the PCBP2 binding for the sB-loop mutants compared to that for the 3′SP mutants supports the likelihood of the steric hindrance scenario, where the hinge-like motion disrupts the correct positioning of the 3′SP and sB-loop, brings these sites in proximity of the sA helix and makes them unapproachable by the PCBP2. However, our results contradict the previous reports (34), which proposed that the hinge-like motion due to the A•C•U base-triple disruption makes the sB-loop more accessible, increasing the affinity of PCBP2 to the REPLRs. Unlike our results with intact PCBP2 and REPLRs, those studies used the isolated PCBP2 KH1/KH2 and KH3 domains and the isolated sB stem-loop and 3′SP constructs. While how the cleavage of PCBP2 affects its affinity to the REPLR remains to be investigated, our findings suggest that the integrity of the PCBP2 and the enteroviral REPLRs play crucial roles in defining their interactions.

### Anti-sense oligos complementary to the 3′SP abolish enteroviral REPLR–PCBP2 interactions

Our binding measurements showed that the sB loop and 3′SP are required for high-affinity interactions of the intact human PCBP2 with enteroviral REPLRs. Our data also suggested that PCBP2 binds tighter with the 3′SP sequence than the sB loop. To further explore the structural features of the 3′SP required for the PCBP2 interactions, we performed PCBP2–REPLR binding experiments in the presence of an antisense DNA oligo (ASO) complementary to the 3′SP sequence for both CVB3 and RVB14 REPLRs. Surprisingly, pre-incubation of the CVB3–3′SP construct with the corresponding ASO (CVB3–3′SP_ASO_, see Supplementary Table S1 for the sequence) that hybridizes with the 3′SP sequence (Figure 5F) abolished the binding with PCBP2 in a dose-dependent manner (Figure 5G). We observed a similar trend of the PCBP2 binding (Figure 5H) for the RVB14–3′SP1 construct pre-incubated with its corresponding ASO (RVB14–3′SP1_ASO_, see Supplementary Table S1 for the sequence). Next, we also performed titration of the corresponding ASOs into the pre-formed CVB3–3′SP–PCBP2 or RVB14–3′SP1–PCBP2 complex (Figure 5I). Interestingly, we observed that the hybridization of ASOs to the 3′SP displaced the PCBP2 from these complexes (Figure 5J, K). While it is unknown whether the 3′SP would engage in the tertiary interactions in the context of the intact 5′ UTR, these results suggest that the enteroviral 3′SP sequences remain single-stranded to interact with PCBP2, which agrees well with the previously reported results for the PCBP2 binding with the isolated 3′SP sequences (34). Moreover, the consistent results for the PCBP2 binding with both CVB3–3′SP and RVB14–3′SP1 construct strongly support that these structural features of the 3′SP sequences are also conserved across the enteroviral replication platforms. As the disruption of the PCBP2 interactions with the enteroviral REPLR has been shown to inhibit viral replication (53), such interventions through ASO could lead to the development of therapeutics against these viruses.

## Discussion

### Conservation of the REPLR’s H-shaped topology and sC–sD interaction across enteroviruses

**Figure 6.**
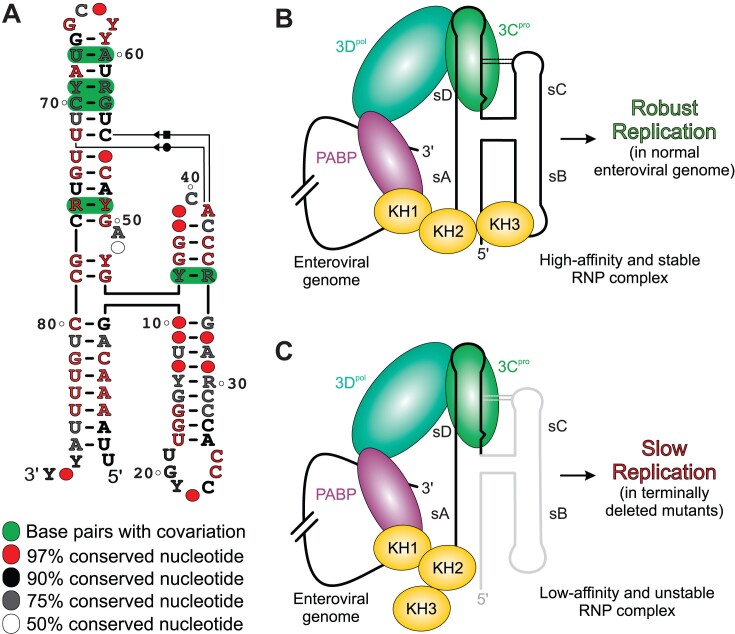
Proposed model of enteroviral genome replication in normal and the 5′ terminally-deleted variants. (**A**) A consensus structure of enteroviral REPLRs based on the seven representative species, including enterovirus A–D and rhinovirus A–C. (**B**) a model showing a REPLR-mediated RNP complex assembly in normal genomes that forms a high-affinity ternary complex, leading to a robust (–)-strand RNA synthesis. (**C**) A model showing a REPLR-mediated RNP complex assembly in terminally-deleted variants (as indicated by the faded gray color) that still could form a low-affinity ternary complex, leading to a slower (–)-strand RNA synthesis implicated in persistent infections.

We previously determined the first high-resolution crystal structure of the CVB3 REPLR using Fab BL3–6 as a crystallization chaperone, where the Fab-binding motif replaced the wild-type sB loop. Later, Gottipati *et al.* (34) reported the CVB3 and PV REPLR structures. Although these two REPLRs were crystallized using a tRNA scaffold appended to the sA, they had almost identical topology as our CVB3 structure, suggesting that the crystallization chaperones (Fab or tRNA scaffold) did not influence the REPLR conformation. The CVB3 REPLR crystal structure reported here with the Fab-binding motif engineered in the sD loop also exhibits the same topology, supporting that the enteroviral REPLRs have modular subdomain structures with the sB and sD loops distal to each other, not favoring tertiary interactions between these loops. Although all enteroviral species were proposed to adopt a cloverleaf-like secondary structure, the primary sequence and predicted local secondary structural features vary among these species. For example, RVB14 is expected to form an sD triloop (UAU), and RVC15 and PV1 show sC pentaloops (CACGU) compared to a typical sD tetraloop (UNCG type, N = A, U, G or C) and sC triloop (NCA, N = A, U, G or C) found in other enteroviral REPLRs. Yet, the last adenosine within the sC loop (A40, A35, A38, in the CVB3, RVB14 and RVC15 crystallization constructs, respectively) is absolutely conserved across all enteroviral REPLRs. Similarly, the sB helix of some REPLRs, such as in RVB14, is shorter than others, such as in CVB3 and RVC15. However, despite these sequence and secondary structure differences, CVB3, RVB14 and RC15 REPLRs crystal structures showed the same consensus H-shaped topology with the sC and sD tertiary interactions through an A•C•U base-triple formation (Figure 6A). Such conserved tertiary interactions observed in our crystal structures also allowed us to compute the structures of EV71, PV1, RVA2 and EVD68 REPLRs using homology modeling, which generated models structurally very similar to our crystal structures. Notably, the *de novo* or homology modeling using other predicted structural features failed to generate the same H-shaped topology of these REPLRs, emphasizing the value of the experimental structures to enrich the RNA structural database and advance RNA structure prediction algorithms.

### Structural basis for REPLR-mediated enteroviral genome replication and translation switch

Human PCBP2 consists of three KH domains (KH1, KH2 and KH3), each separated by flexible linkers with a much longer linker between the KH2 and KH3 domains (54). It is known to recognize the two distinct regions within the 5′ UTR of enteroviruses, the REPLR (domain I) and IRES domain IV, where its binding to the REPLR promotes genome replication and binding to the IRES domain IV promotes the translation (17,20,27,35), leading to a hypothesis that PCBP2 may regulate the switching between the enteroviral genome replication and translation. Based on the arrangement of PCBP2 and 3CD binding sites within the REPLRs, Gottipati *et al.* (34) proposed that whereas a weaker binding of multiple PCBP2 to the REPLR leads to the translation, a high-affinity binding in the presence of the 3CD promotes the replication. In this proposed mechanism, the 3CD binding to the sD induces a global conformational change in the REPLR structure, allowing a hinge-like motion and disrupting the sC–sD tertiary interaction, which exposes the sB loop for the high-affinity binding. However, this model relied heavily on the results from isolated PCBP2 KH1 and KH2/KH3 domains and REPLR sB stem–loop and 3′SP sequences. While some results are consistent with our studies using intact PCBP2 and REPLR constructs, their overall replication-translation switching model does not support why the disruption of the A•C•U base-triple diminishes the PCBP2 binding. We have proposed a new REPLR-mediated enteroviral replication and translation switching model based on our studies with full-length PCBP2 protein and intact enteroviral REPLR constructs with the 3′-spacers (Figure 6B).

When the (+)-strand enteroviral genome is released into the host cytoplasm, the PCBP2 can bind to both the REPLR and the domain IV, with a relatively higher affinity for the domain IV compared to the REPLR (17), allowing efficient translation of the genome to produce viral proteins. Once viral proteins are sufficiently available, 3C protease cleaves the PCBP2 bound to domain IV to shut off the translation, and 3CD binds the REPLR, increasing the affinity of the PCBP2 to the REPLR. Then, this ternary complex interacts with the poly(A)–PABP complex at the 3′ end to circularize the viral genome. In this RNP complex (Figure 6B), the modular and pre-organized H-shaped structure brings the sB loop and 3′SP to bind a single copy of the intact PCBP2, consistent with the highest affinity of the REPLR-3′SP compared to the corresponding sB, 3′SP or both mutant constructs. Any disruption to the integrity of the H-shaped REPLR structure stabilized by sC–sD tertiary interaction compromises the affinity of PCBP2 binding and, thus, the (–)-strand synthesis. Moreover, 3CD has to be auto-cleaved to free up the 3D polymerase to initiate the RNA synthesis catalysis. While this warrants further studies, the released 3C protease may cleave the PCBP2, thus weakening the PCBP2 affinity to the REPLR, consistent with weaker binding of the REPLR–3′SP constructs with the isolated KH1 and KH2/KH3 domains compared to that of the intact PCBP2 (34). As the binding of the 3CD to the REPLR is known to downregulate the genome translation (55), we assume that cleavage of the 3CD or PCBP2 or both perhaps helps disassemble the replication-competent REPLR complex, abolishing the replication and facilitating the (+)-strands towards the translation. Nevertheless, whether the same (+)-strand undergoes multiple replication and translation cycles remains to be investigated.

### Structural basis for 3CD and PCBP2 binding in the clinically isolated enteroviral REPLRs

Previous studies have shown that clinical isolates of enteroviruses from patients with myocarditis and dilated cardiomyopathy contain various nucleotide deletions (7–49 nts) from the 5′ end of the viral genome (56). While the exact mechanisms and functions of such deletions are unknown, the viruses with these deletions have been shown to replicate slowly, causing persistent infection. Structurally, the most significant deletion (49 nts) disrupts the double-stranded sA and completely removes the sB and sC of the REPLRs, suggesting that the sD is the minimal structural motif required for the enteroviral (–)-strand synthesis. Interestingly, our studies revealed that isolated sD (stem-loop) binds to the 3C with similar affinity as the intact enteroviral REPLR (36), illuminating the structural basis for the 3CD recruitment during (–)-strand synthesis in the terminally deleted genomes (Figure 6C). Moreover, with the high-affinity PCBP2 binding site (3′SP) being intact, the 3CD-PCBP2 ternary interactions are likely to be preserved even without the sB loop (but with low-affinity of PCBP2 binding), supporting the (–)-strand synthesis. Nevertheless, the affinity of PCBP2 binding is highest with the intact REPLRs, explaining the much faster replication of the non-deleted genomes compared to the deleted variants (14,57).

Furthermore, only the (+)-strand genomic RNA, when present in a large excess compared to the (–)-strands, are packaged into the new infectious virions. However, the terminal deletions in enteroviral genomes also decreased significantly the (–)- to (+)-strand ratio (almost 1:1 in some cases versus the typical 1:12 molar ratio), leading to even the packaging of (–)-strand in the virions, indicating that these deletions were more detrimental for the (+)-strand synthesis than for the (–)-strand synthesis (14). Nevertheless, we also found that the affinity of PCBP2, not the 3C, for intact enteroviral REPLRs is significantly reduced when a single mutation disrupts the sC–sD interactions. Although the effects of the sC–sD disruption on the viral genome replication remain to be studied comprehensively, the destabilization of the H-shaped structure by disrupting the sC–sD tertiary interactions is likely to affect the (+)-strand synthesis deleteriously, similar to that observed for those deletion variants (14). While several structural features are conserved across the enteroviral REPLRs, including the sD loop, dinucleotide bulge, Py–Py helix and the sC–sD tertiary interaction, taken together with the previous *in vivo* functional observations (14), our studies suggest that the structural integrity of the enteroviral REPLRs are perhaps more sensitive to the efficient synthesis of (+)-strands rather than the (–)-strands. Although further structural and mechanistic studies of enteroviral REPLRs formed within the 3′ termini of the (–)-strands are needed to understand the overall enteroviral replication mechanisms better, our study provides a structural basis for the (–)-strand synthesis during replication of wild-type enteroviral genomes as well as the terminally deleted variants observed in several clinical isolates, tissue, and primary cell cultures.

### Enteroviral REPLRs as potential therapeutic targets

Disrupting the RNA-protein complex formation within the enteroviral REPLRs affects viral replication, and thus, such interventions could lead to the development of therapeutics against these viruses. Our studies revealed that the ASOs that bind to the 3′SP sequences within the enteroviral replication platform abrogate the PCBP2 binding, directly impacting the critical step of genome replication. As 3′SP sequences represent a highly conserved region in enteroviral genomes, and ASOs have been successfully used previously to treat several other diseases (58–60), our studies highlight potential applications of ASO-based therapeutics against enteroviruses targeting this platform. Our high-resolution crystal structures also provide opportunities for structure-based screening and designing small-molecule drugs. Notably, given the high conservation of REPLRs, such small molecules can be universal drugs against multiple enteroviruses.

## Supplementary Material

gkae627_Supplemental_File

## Data Availability

Atomic coordinates and structure factors for the reported crystal structures of CVB3–DL_mut_, RVB14–DL_mut_, RVC15–DL_mut_ and RVC15–BL_mut_ constructs have been deposited with the Protein Data Bank under the accession codes 8VM8, 8VM9, 8VMA and 8VMB, respectively. Upon a reasonable request, the authors will provide the raw data, additional information, and materials, including the plasmid for Fab BL3–6 expression. The requests should be addressed to D.K.
