# Supplementary material for: Structural basis for a highly conserved RNA-mediated enteroviral genome replication
Source: Nucleic Acids Res. 2024 Jul 22;52(18):11218–33. doi: 10.1093/nar/gkae627 (PMC11472160; doi:10.1093/nar/gkae627)
Supplement: gkae627_Supplemental_File [file gkae627_supplemental_file.pdf]

Supplementary Information

Structural basis for a highly conserved RNA-mediated enteroviral genome replication

Naba Krishna Das, Jeff Vogt, Alisha Patel, Hasan Al Banna, Deepak Koirala*

Department of Chemistry and Biochemistry, University of Maryland Baltimore County, Baltimore, Maryland 21250, USA

*To whom correspondence should be addressed

dkoirala@umbc.edu

Supplementary Table S1. The sequences of RNAs, DNA ASOs, and protein constructs that were used in this study.

Constructs	Sequence
EV71$_{WT}$	5'GGUAAAACAGCCUGUGGGUUGCACCCACUCACAGGGCCCAC UGGGCGCAAGCACUCUGGUACCUCGGUACCUUUGUGCGCCU GUUUUACC
EV71-BL$_{mut}$	5'GGUAAAACAGCCUGUGGGUGAAACACACUCACAGGGCCCAC UGGGCGCAAGCACUCUGGUACCUCGGUACCUUUGUGCGCCU GUUUUACC
EV71-DL$_{mut}$	5'GGUAAAACAGCCUGUGGGUUGCACCCACUCACAGGGCCCAC UGGGCGCAAGCACUCUGGUACGAAACACGUACCUUUGUGCGC CUGUUUUACC
CVB3$_{WT}$	5'GGUAAAACAGCCUGUGGGUUGAUCCCACCCACAGGGCCCAU UGGGCGCUAGCACUCUGGUAUCACGGUACCUUUGUGCGCCU GUUUUACC
CVB3-BL$_{mut}$	5'GGUAAAACAGCCUGUGGGUGAAACACACCCACAGGGCCCAU UGGGCGCUAGCACUCUGGUAUCACGGUACCUUUGUGCGCCU GUUUUACC
CVB3-DL$_{mut}$	5'GGUAAAACAGCCUGUGGGUUGAUCCCACCCACAGGGCCCAU UGGGCGCUAGCACUCUGGUAUGAAACACGUACCUUUGUGCG CCUGUUUUACC
CVB3-3'SP$_{AA}$	5'GGUAAAACAGCCUGUGGGUUGAUCCCACCCACAGGGCCCAU UGGGCGCUAGCACUCUGGUAUCACGGUACCUUUGUGCGCCU GUUUUACUACCCCCUCCCCCAA
CVB3-DL$_{mut}$-3'SP$_{AA}$	5'GGUAAAACAGCCUGUGGGUUGAUCCCACCCACAGGGCCCAU UGGGCGCUAGCACUCUGGUAUGAAACACGUACCUUUGUGCG CCUGUUUUACUACCCCCUCCCCCAA
CVB3-3'SP	5'GGUAAAACAGCCUGUGGGUUGAUCCCACCCACAGGGCCCAU UGGGCGCUAGCACUCUGGUAUCACGGUACCUUUGUGCGCCU GUUUUACUACCCCCUCCCCC
CVB3-3'SP2	5'GUAAAACAGCCUGUGGGUUGAUCCCACCCACAGGGCCCAUU GGGCGCUAGCACUCUGGUAUCACGGUACCUUUGUGCGCCUG UUUUAUACCCCCUCCCCC
CVB3-3'SP$_{A40U}$	5'GGUAAAACAGCCUGUGGGUUGAUCCCACCCACAGGGCCCUU UGGGCGCUAGCACUCUGGUAUCACGGUACCUUUGUGCGCCU GUUUUACUACCCCCUCCCCC
CVB3-3'SP-DL$_{mut}$	5'GGUAAAACAGCCUGUGGGUUGAUCCCACCCACAGGGCCCAU UGGGCGCUAGCACUCUGGUAUGAAACACGUACCUUUGUGCG CCUGUUUUACUACCCCCUCCCCC
CVB3-3'SP-BL$_{mut}$	5'GGUAAAACAGCCUGUGGGUUGAUCUUACCCACAGGGCCCAU UGGGCGCUAGCACUCUGGUAUGAAACACGUACCUUUGUGCG CCUGUUUUACUAC CCCUCCCCC
CVB3-3'SP$_{mut}$	5'GGUAAAACAGCCUGUGGGUUGAUCCCACCCACAGGGCCCAU UGGGCGCUAGCACUCUGGUAUGAAACACGUACCUUUGUGCG CCUGUUUUACUACUCUCUCUCUCAA

CVB3-3'SP$_{mut}$BL$_{mut}$	5'GGUAAAACAGCCUGUGGGUUGAUCUUACCCACAGGGCCCAUUGGGCGCUAGCACUCUGGUAUGAAACACGUACCUUUGUGCGCCUGUUUUACUACUCUCUCUCUCAA
CVB3-3'SP$_{HP}$	5'GGUAAAACAGCCUGUGGGUUGAUCCCACCCACAGGGCCCAUUGGGCGCUAGCACUCUGGUAUCACGGUACCUUUGUGCGCCUGUUUUACUACCCCCUCCCCCAACUGUAACCGAAAGGUUAC
PV1$_{WT}$	5'GGUAAAACAGCUCUGGGGUUGUACCCACCCCAGAGGCCCACGUGGCGGCUAGUACUCCGGUAUUGCGGUACCCUUGUACGCCUGUUUUACC
PV1-BL$_{mut}$	5'GGUAAAACAGCUCUGGGGUGAAACACACCCCAGAGGCCCACGUGGCGGCUAGUACUCCGGUAUUGCGGUACCCUUGUACGCCUGUUUUACC
PV1-DL$_{mut}$	5'GGUAAAACAGCUCUGGGGUUGUACCCACCCCAGAGGCCCACGUGGCGGCUAGUACUCCGGUAUGAAAGACGUACCCUUGUACGCCUGUUUUACC
EVD68$_{WT}$	5'GGUUAAAACAGCUCUGGGGUUGUUCCCACUCAAGGGCCCACGUGGCGGCUAGUACUCUGGUAUCUCGGUACCUUUGUACGCCUGUUUUAACC
EVD68-BL$_{mut}$	5'GGUUAAAACAGCUCUGGGGGAAACACUCCCACUCAAGGGCCCACGUGGCGGCUAGUACUCUGGUAUCUCGGUACCUUUGUACGCCUGUUUUAACC
EVD68-DL$_{mut}$	5'GGUUAAAACAGCUCUGGGGUUGUUCCCACUCAAGGGCCCACGUGGCGGCUAGUACUCUGGUAUGAAACACGUACCUUUGUACGCCUGUUUUAACC
RVA2$_{WT}$	5'GGUAAAACUGGAUCCAGGUUGUUCCCACCUGGAUUUCCCACAGGGAGUGGUACUCUGUUAUUACGGUAACUUUGUACGCCAGUUUUACC
RVA2-BL$_{mut}$	5'GGUAAAACUGGAUCCAGGUGAAACACACCUGGAUUUCCCACAGGGAGUGGUACUCUGUUAUUACGGUAACUUUGUACGCCAGUUUUACC 3'
RVA2-DL$_{mut}$	5'GGUAAAACUGGAUCCAGGUUGUUCCCACCUGGAUUUCCCACAGGGAGUGGUACUCUGUUAUGAAACACGUAACUUUGUACGCCAGUUUUACC
RVB14$_{WT}$	5'GGAAACAGCGGAUGGGUAUCCCACCAUUCGACCCAUUGGGUGUAGUACUCUGGUACUAUGUACCUUUGUACGCCUGUUUCC
RVB14-BL$_{mut}$	5'GGAAACAGCGGAUGGGAAACACCCAUUCGACCCAUUGGGUGUAGUACUCUGGUACUAUGUACCUUUGUACGCCUGUUUCC
RVB14-DL$_{mut}$	5'GGAAACAGCGGAUGGGUAUCCCACCAUUCGACCCAUUGGGUGUAGUACUCUGGUACGAAACACGUACCUUUGUACGCCUGUUUCC
RVB14-3'SP1	5'GGAAACAGCGGAUGGGUAUCCCACCAUUCGACCCAUUGGGUGUAGUACUCUGGUACUAUGUACCUUUGUACGCCUGUUUCUCCCCAACCA CCCUU
RVB14-3'SP1-DL$_{mut}$	5'GGAAACAGCGGAUGGGUAUCCCACCAUUCGACCCAUUGGGUGUAGUACUCUGGUACGAAACACGUACCUUUGUACGCCUGUUUCUCCCCAACCACCCUU

RVB14-3'SP1$_{A35U}$	5'GGAAACAGCGGAUGGGUAUCCCACCAUUCGACCCUUUGGGU GUAGUACUCUGGUACUAUGUACCUUUGUACGCCUGUUUCUCC CCAACCACCCUU
RVB14-3'SP1-BL$_{mut}$	5'GGAAACAGCGGAUGGGUAUCUUACCAUUCGACCCAUUGGGU GUAGUACUCUGGUACUAUGUACCUUUGUACGCCUGU UUCUCCCCAACCACCCUU
RVB14-3'SP1$_{mut}$	5'GGAAACAGCGGAUGGGUAUCCCACCAUUCGACCCAUUGGGU GUAGUACUCUGGUACUAUGUACCUUUGUACGCCUGU UUCUCUCUAACUACUCUU
RVB14-3'SP1$_{mut}$BL$_{mut}$	5'GGAAACAGCGGAUGGGUAUCUUACCAUUCGACCCAUUGGGU GUAGUACUCUGGUACUAUGUACCUUUGUACGCCUGUUUCUCU CUAACUACUCUU
RVB14-3'SP2	5'GGAAACAGCGGAUGGGUAUCCCACCAUUCGACCCAUUGGGU GUAGUACUCUGGUACUAUGUACCUUUGUACGCCUGUUUCUCC CCAACCACCCUUCCUUAAAAUUCCCACCC
RVC15$_{WT}$	5'GGAAACUGGGUAUAGGUUGUUCCCACCUGUACCACCCACGU GGUGUAGUGCUCCUGUAUUCCGGUACACUUGCACGCCAGUU UCC
RVC15-BL$_{mut}$	5'GGAAACUGGGUAUAGGUGAAACACACCUGUACCACCCACGU GGUGUAGUGCUCCUGUAUUCCGGUACACUUGCACGCCAGUU UCC
RVC15-DL$_{mut}$	5'GGAAACUGGGUAUAGGUUGUUCCCACCUGUACCACCCACGU GGUGUAGUGCUCCUGUAUGAAACACGUACACUUGCACGCCAG UUUCC
CVB3-3'SP-ASO	5'GGGGGAGGGGGTA
RVB14-3'SP1-ASO	5'AAGGGTGGTTGGGG
Human PCBP2 (residues 11-359)	MNVTLTIRLLMHGKEVGSIIGKKGESVKKMREESGARINISEGNCP ERIITLAGPTNAIFKAFAMIIDKLEEDISSSMTNSTAASRPPVTLRLVV PASQCGSLIGKGGCKIKEIRESTGAQVQVAGDMLPNSTERAITIAGI PQSIIECVKQICVVMLETLSQSPPKGVTIPYRPKPSSSPVIFAGGQD RYSTGSDSASFPHTTPSMCLNPDLEGPPLEAYTIQGQYAIPQPDLT KLHQLAMQQSHFPMTHGNTGFSGIESSSPEVKGYWGLDASAQTT SHELTIPNDLIGCIIGRQGAKINEIRQMSGAQIKIANPVEGSTDRQVTI TGSAASISLAQYLINVRLSSETLEHHHHHH

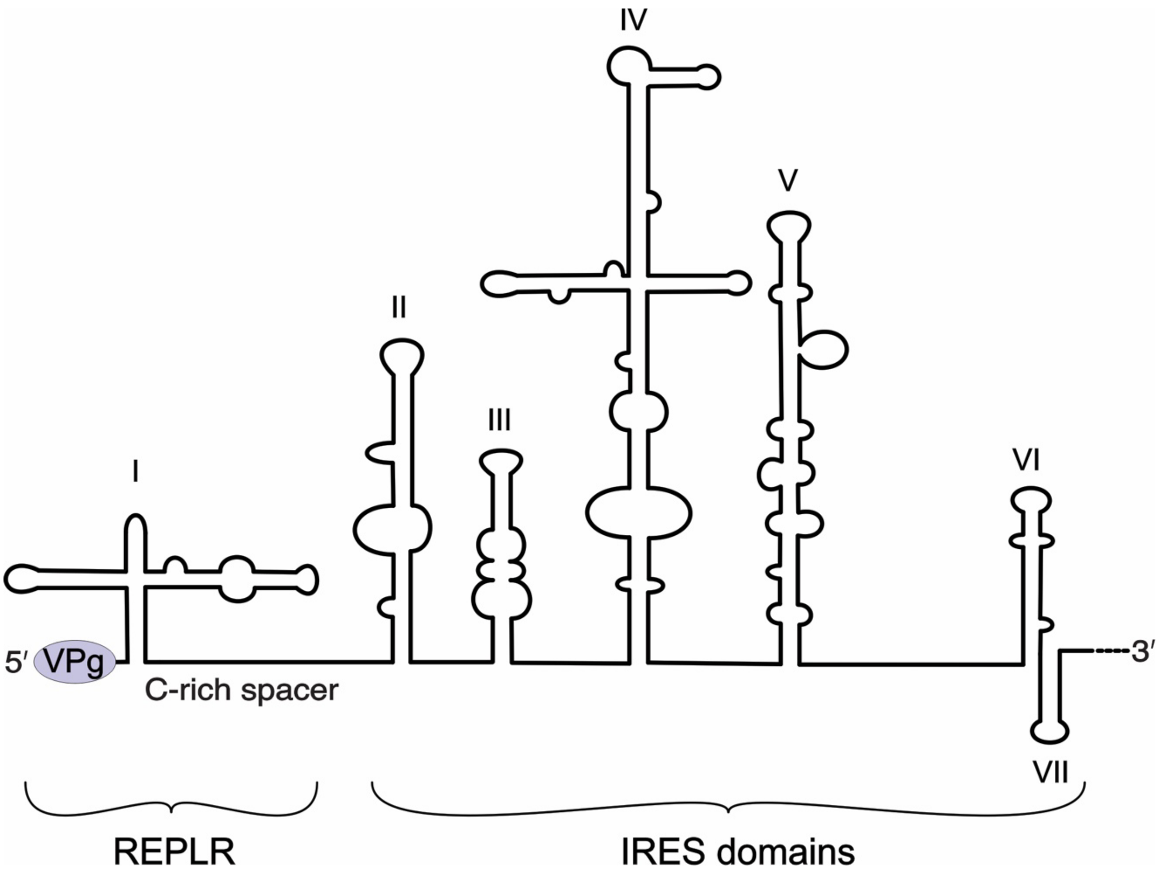

Supplementary Figure S1: Schematic illustration of the modular RNA domains within the 5′ untranslated region (UTR) of enteroviral genomes. The predicted secondary structural model of the CVB3 5′ UTR, according to Bailey *et al.* (1), is shown here. Domain I, with a C-rich spacer sequence in its 3′ side, represents the replication-linked RNA (REPLR) platform, which promotes the (-)-strand RNA synthesis during genome replication. Domains II to VII comprise the internal ribosome entry site (IRES), which drives the translation of genome-encoded viral proteins.

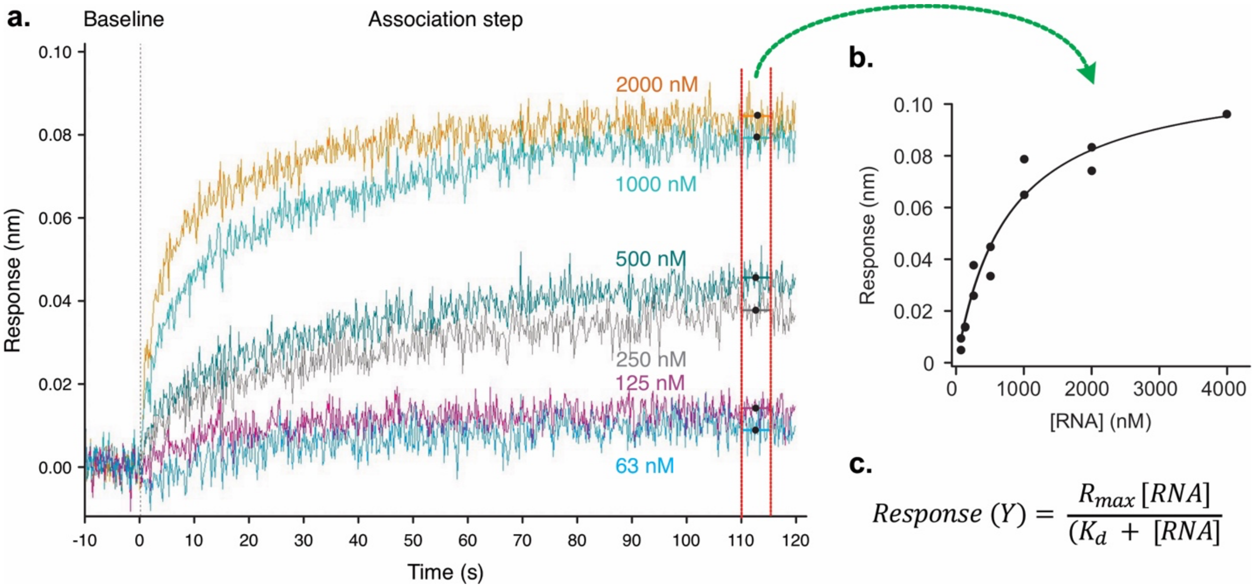

Supplementary Figure S2: The BLI-based measurements of the PCBP2 binding interactions with enteroviral REPLRs. (a) Representative BLI responses as a function of time when the PCBP2-immobilized biosensors were incubated with the various concentrations of REPLR constructs. BLI response-time traces shown here are after the reference subtraction and baseline alignment using the BLI system-integrated software. (b) Average BLI responses from the plateau region (110 – 115 seconds, as indicated by the dotted vertical red lines) were calculated and plotted against RNA concentration (filled circles). Data were fitted with a binding isotherm (solid curve), as shown in panel c.

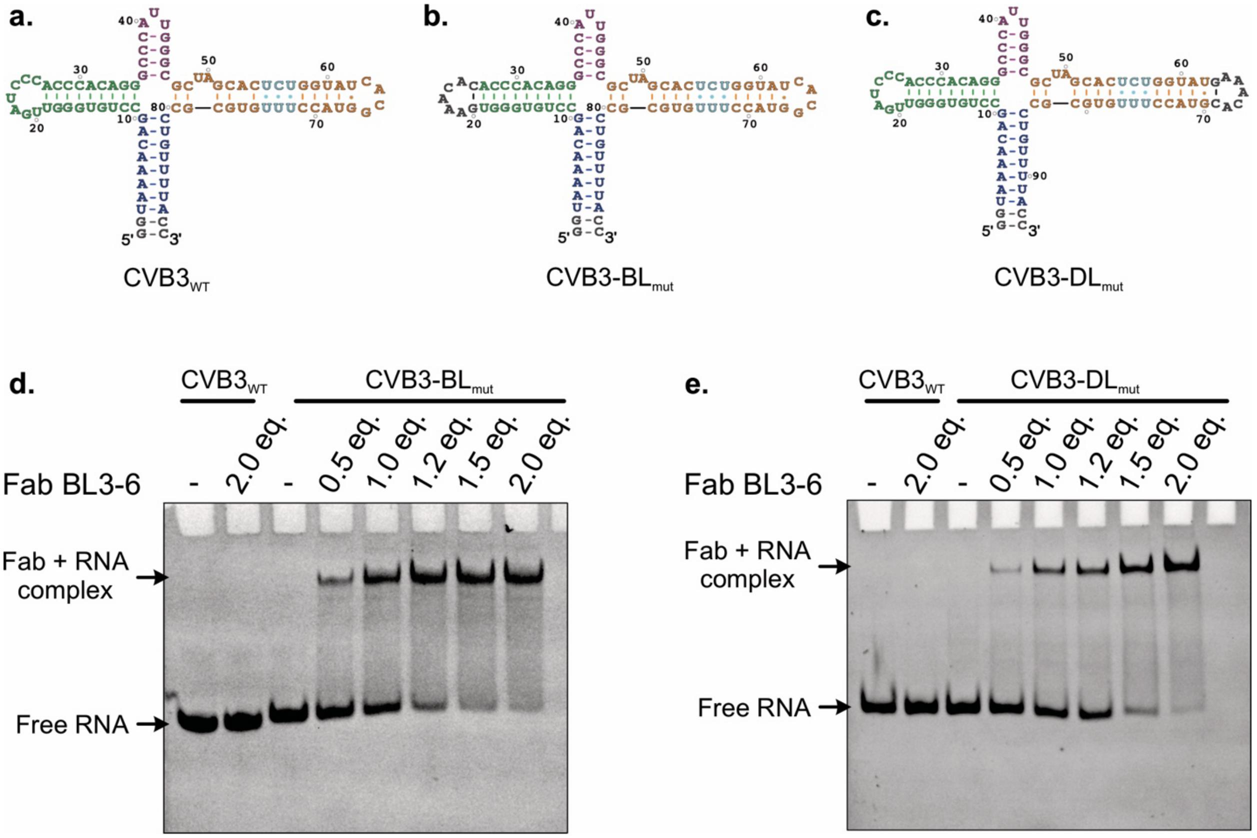

Supplementary Figure S3: The CVB3 REPLR crystallization constructs and Fab binding. (a) The wild-type REPLR construct includes the two additional G-C pairs (colored gray) closing the sA helix. The crystallization constructs with the (b) sB-loop (CVB3-BL_{mut}) and (c) sD-loop (CVB3-DL_{mut}) mutation to create a Fab BL3-6 binding site (5′ GAAACAC sequence, gray). The native polyacrylamide gel electrophoresis (nPAGE) shows the similar binding of Fab BL3-6 with the (d) CVB3-BL_{mut} and (e) CVB3-DL_{mut} constructs. Each lane was loaded with 100 ng of RNA. The wild-type (without Fab binding motif) served as a negative control. The slower migrating Fab-RNA complex bands appeared only for the constructs with the Fab binding motif.

Supplementary Table S2. X-ray crystallography data collection and structure refinement statistics. The values in parentheses are for the highest-resolution shell.

**Data collection**	**CVB3-DL$_{mut}$ (PDB: 8VM8)**	**RVB14-DL$_{mut}$ (PDB: 8VM9)**	**RVC15-DL$_{mut}$ (PDB: 8VMA)**	**RVC15-BL$_{mut}$ (PDB: 8VMB)**
Space group	C121	P12$_1$1	P12$_1$1	I2$_1$2$_1$2$_1$
Resolution (Å)	38.31 - 1.54 (1.57 - 1.54)	139.63 - 2.20 (2.24 - 2.20)	140.97 - 2.54 (2.62 - 2.54)	99.93 – 2.97 (3.15 – 2.97)
Cell dimensions				
a, b, c (Å)<br>α, β, γ (°)	145.99, 80.37, 87.34<br>90, 111.17, 90	70.92, 79.21, 141.32<br>90, 98.87, 90	52.99, 106.37, 142.82<br>90, 99.23, 90	103.22, 121.85, 174.66<br>90, 90, 90
R_{merge} (%)	4.9 (189.4)	5.0 (79.1)	6.2 (112.8)	5.5 (166.9)
I/σI	13.7 (0.7)	11.2 (1.4)	12.3 (1.0)	19.2 (0.9)
$CC_{1/2}$	0.999 (0.348)	0.997 (0.662)	0.999 (0.425)	1.000 (0.643)
Completeness (%)	99.8 (99.8)	98.3 (94.8)	99.5 (99.6)	99.8 (99.4)
Redundancy	4.2 (4.1)	3.5 (3.5)	3.5 (3.6)	6.8 (6.9)
**Refinement**				
No. reflections	138,691 (6873)	77,582 (4411)	51,456 (4415)	23,068 (3652)
R_{work} – R_{free} (%)	18.6 – 21.3	20.0 – 22.9	20.5 – 24.4	21.0 – 25.1
R.M.S. deviations				
Bond angles (°)	0.764	1.019	1.246	1.150
Bond length (Å)	0.005	0.009	0.010	0.009
Average B-factor, all atoms (Å^2)	55.0	116.0	116.0	137.0
Ramachandran plot of protein residues				
Preferred regions (%)	97.95	96.67	96.44	94.99
Allowed regions (%)	2.05	3.33	3.55	5.01

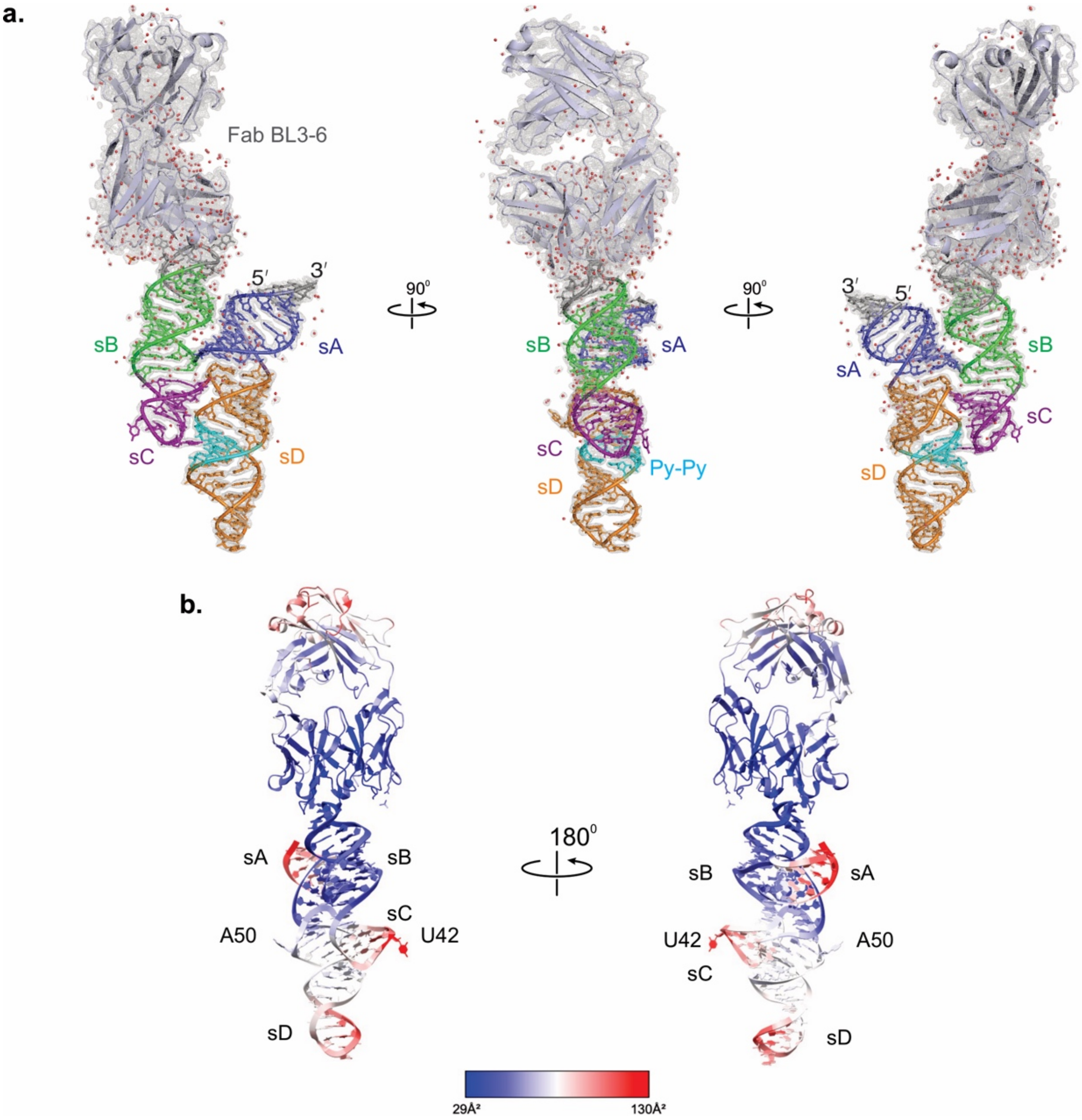

Supplementary Figure S4: Overall crystal structure of the CVB3-BL_{mut} REPLR construct. (a) The crystal structure of the CVB3-BL_{mut} in complex with Fab BL3-6 solved at 1.9 Å resolution, which has been published elsewhere (PDB: 8DP3) (2). The crystals have a single Fab-RNA complex per asymmetric unit. The gray mesh represents the $2|F_o| - |F_c|$ electron density map at 1σ contour level and carve radius 1.8 Å, and the red spheres depict water molecules. (b) The crystal structure of the same complex colored according to the crystallographic B-factors. The gradient from blue to red indicates the lowest (29 Å^2) and the highest (130 Å^2) B-factors.

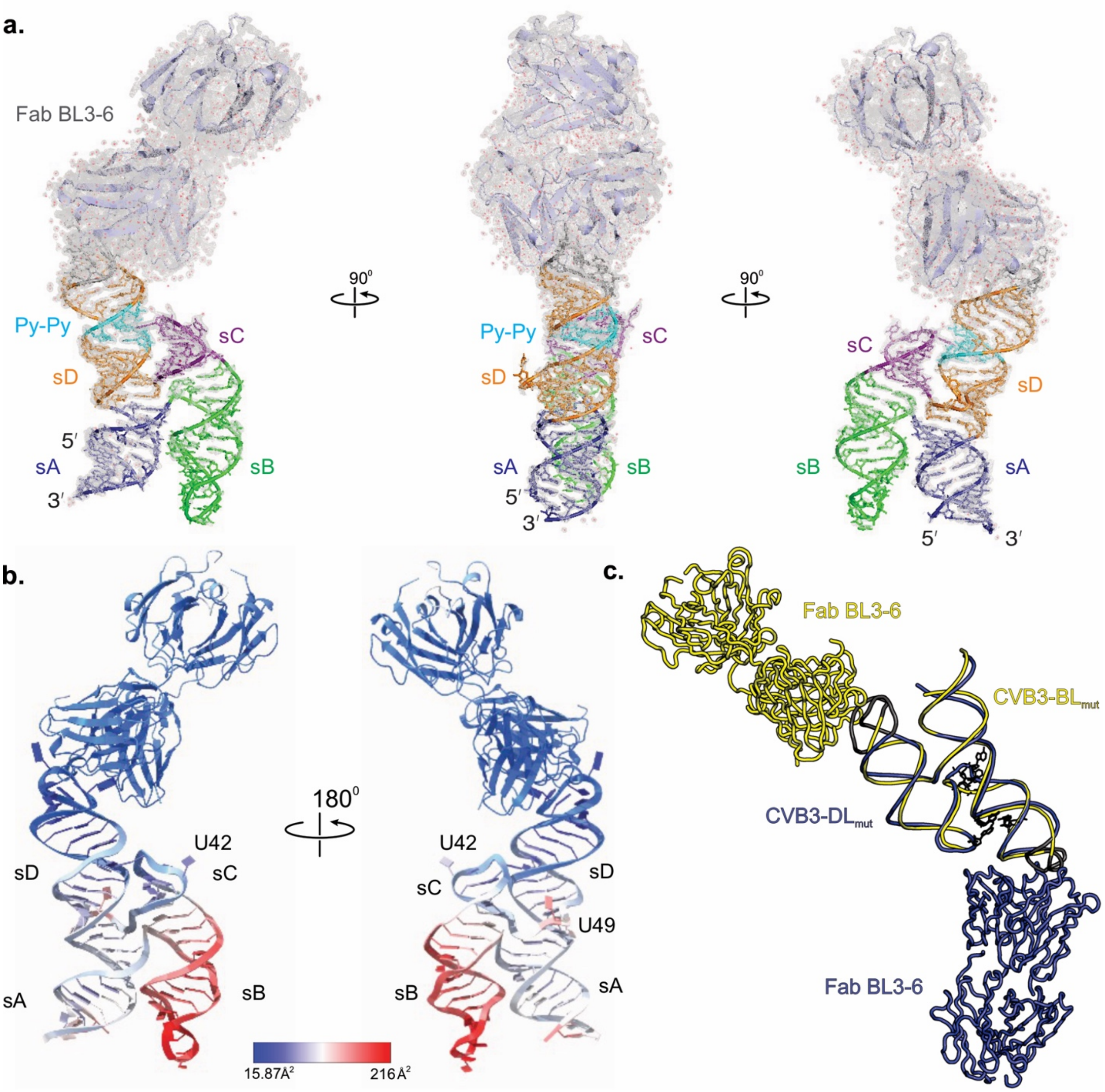

Supplementary Figure S5: Overall crystal structure of the CVB3-DL$_{mut}$ REPLR construct. (a) The crystal structure of the CVB3-DL$_{mut}$ in complex with Fab BL3-6 solved at 1.54 Å resolution (PDB: 8VM8). The asymmetric unit contains a single Fab-RNA complex. The gray mesh represents the $2|F_o| - |F_c|$ electron density map at 1σ contour level and carve radius 1.8 Å, and the red spheres depict water molecules. (b) The crystal structure of the same complex colored according to the crystallographic B-factors, with blue for the lowest (15.87 Å^2) and red for the highest (216 Å^2) B-factors. (c) the superposition of the common core of CVB3-DL$_{mut}$ (blue) and CVB3-BL$_{mut}$ (yellow) crystal structures (RMSD = 1.98 Å) without considering the sB and sD loops (colored gray).

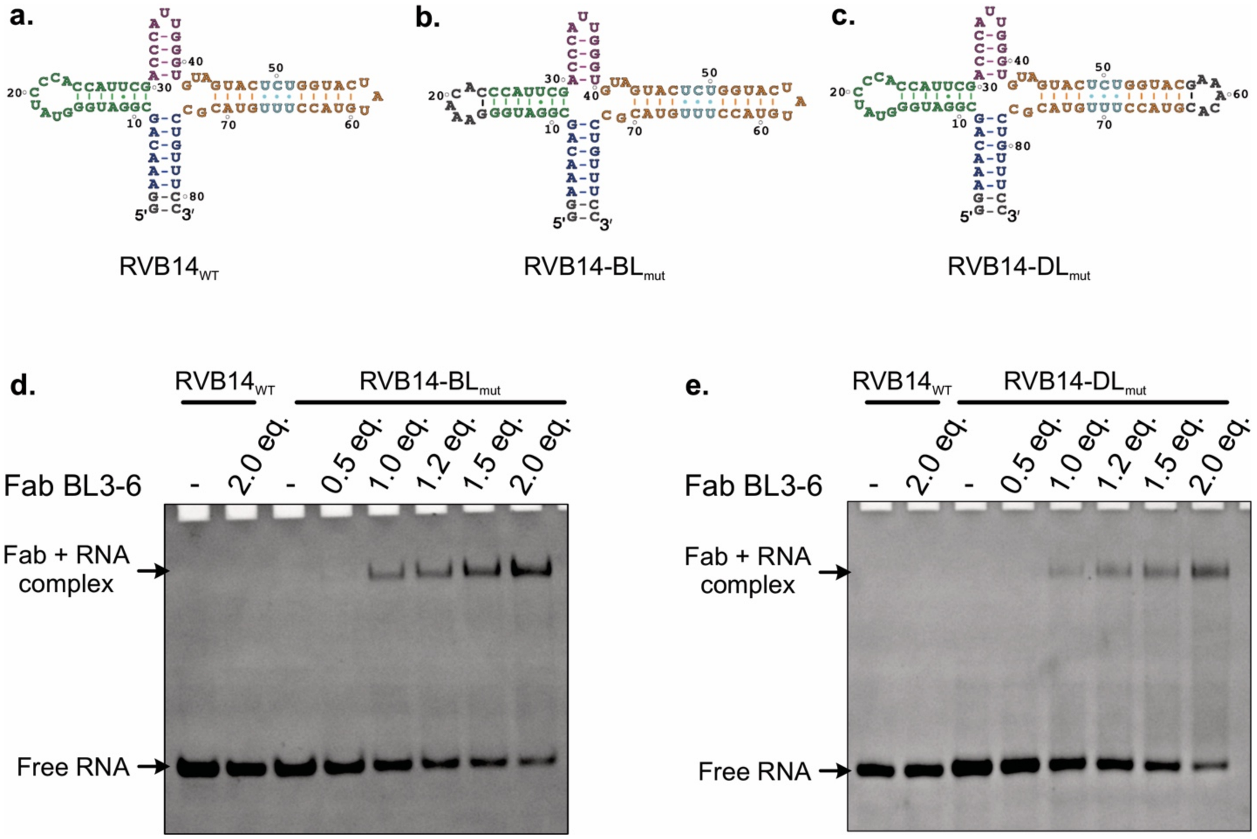

Supplementary Figure S6: The RVB14 REPLR crystallization constructs and Fab binding. (a) The wild-type REPLR construct includes the two additional G-C pairs (colored gray) closing the sA helix. The crystallization constructs with the (b) sB-loop (RVB14-BL$_{mut}$) and (c) sD-loop (RVB14-DL$_{mut}$) mutation to create a Fab BL3-6 binding site (5′ GAAACAC sequence, gray). The native polyacrylamide gel electrophoresis (nPAGE) shows the similar binding of Fab BL3-6 with the (d) RVB14-BL$_{mut}$ and (e) RVB14-DL$_{mut}$ constructs. Each lane was loaded with 100 ng of RNA. The wild-type (without Fab binding motif) served as a negative control. The slower migrating Fab-RNA complex bands appeared only for the constructs with the Fab binding motif.

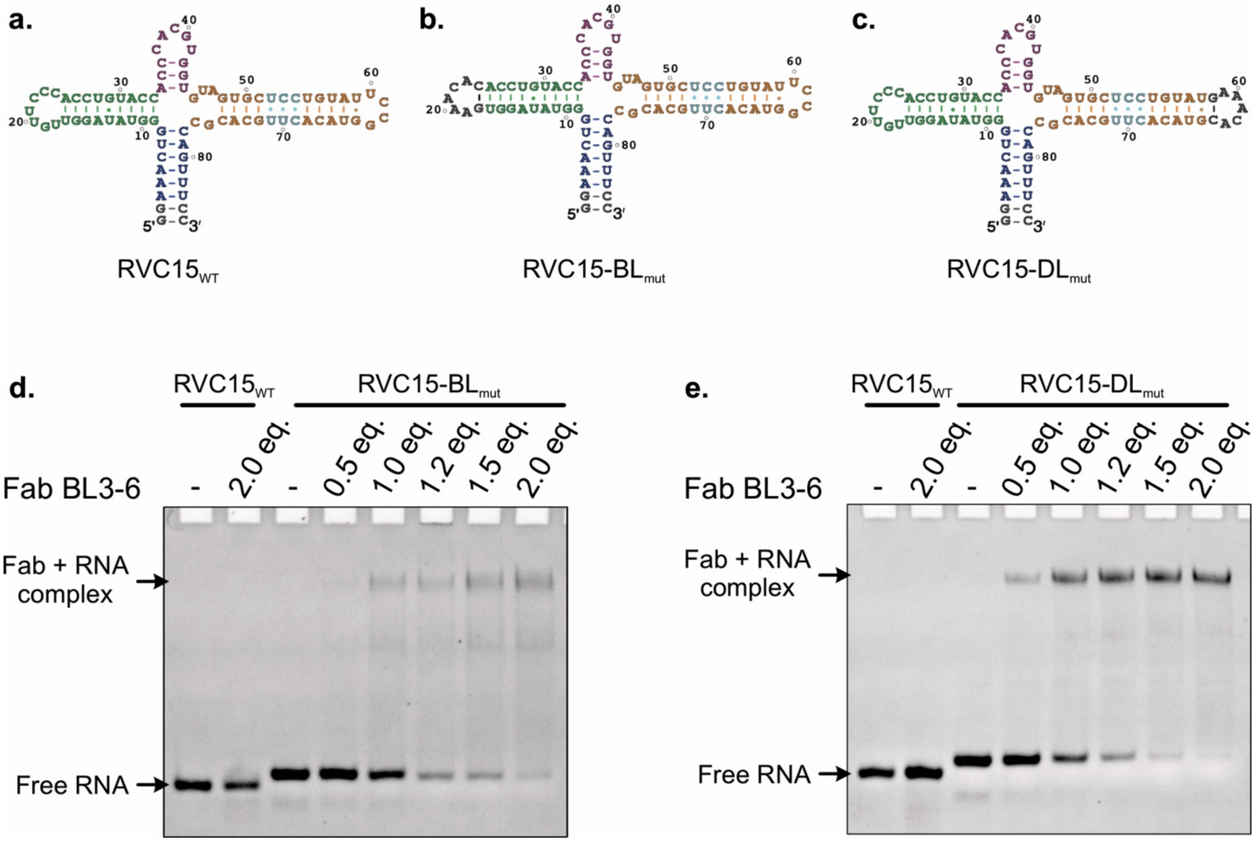

Supplementary Figure S7: The RVC15 REPLR crystallization constructs and Fab binding. (a) The wild-type REPLR construct includes the two additional G-C pairs (colored gray) closing the sA helix. The crystallization constructs with the (b) sB-loop (RVC15-BL$_{mut}$) and (c) sD-loop (RVC15-DL$_{mut}$) mutation to create a Fab BL3-6 binding site (5′ GAAACAC sequence, gray). The native polyacrylamide gel electrophoresis (nPAGE) shows the similar binding of Fab BL3-6 with the (d) RVC15-BL$_{mut}$ and (e) RVC15-DL$_{mut}$ constructs. Each lane was loaded with 100 ng of RNA. The wild-type (without Fab binding motif) served as a negative control. The slower migrating Fab-RNA complex bands appeared only for the constructs with the Fab binding motif.

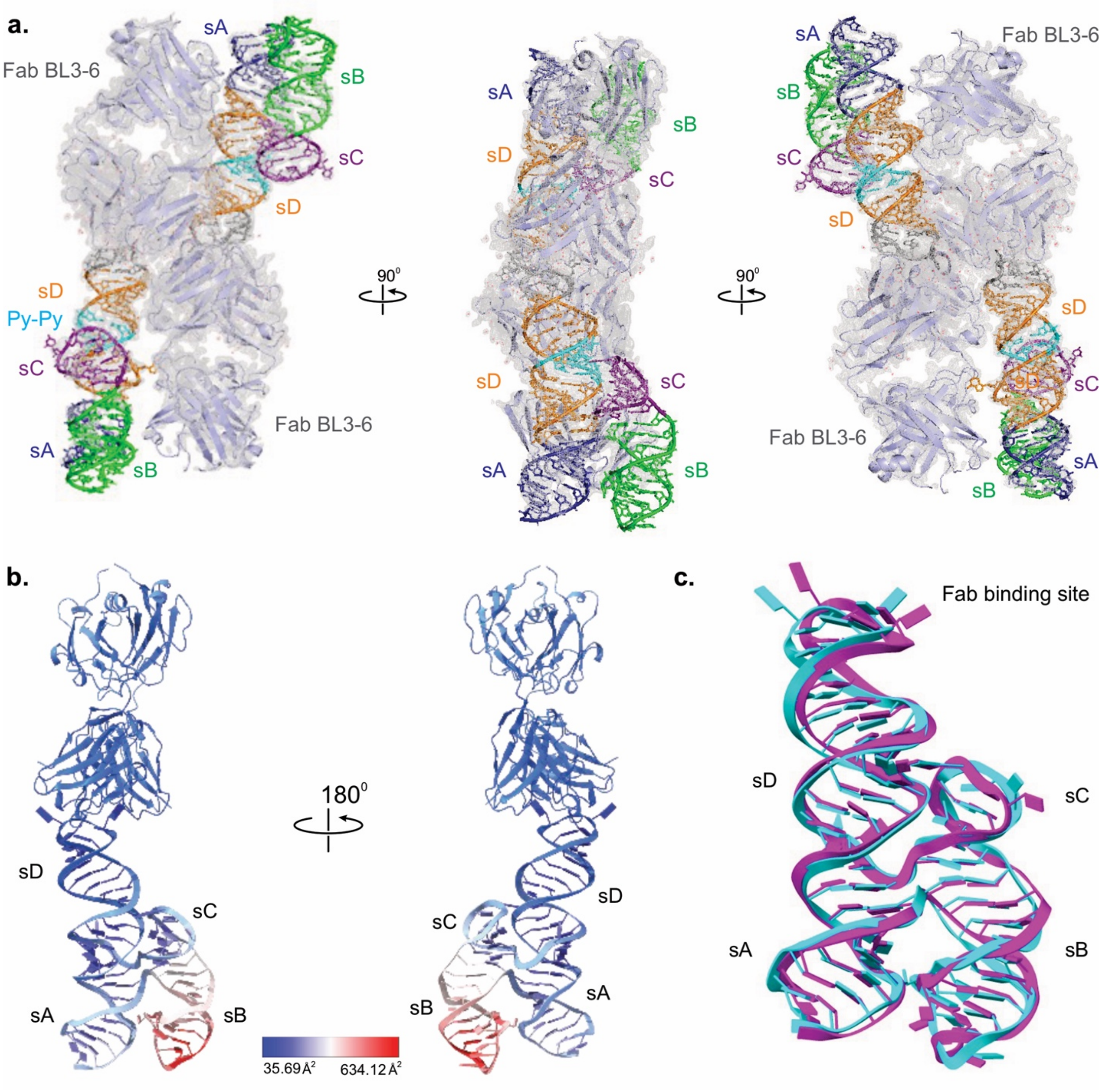

Supplementary Figure S8: Overall crystal structure of the RVB14-DL$_{mut}$ REPLR construct. (a) The crystal structure of the RVB14-DL$_{mut}$ in complex with Fab BL3-6 solved at 2.20 Å resolution (PDB: 8VM9). The crystals have two Fab-RNA complexes per asymmetric unit. The gray mesh represents the $2|F_o| - |F_c|$ electron density map at 1σ contour level and carve radius 1.8 Å, and the red spheres depict water molecules. (b) The crystal structure of the same complex colored according to the crystallographic B-factors, with blue for the lowest (35.69 Å^2) and red for the highest (634.12 Å^2) B-factors. (c) the superposition of the two RVB14-DL$_{mut}$ molecules within the asymmetric unit (RMSD = 1.530 Å). The two molecules are colored cyan and magenta for clarity.

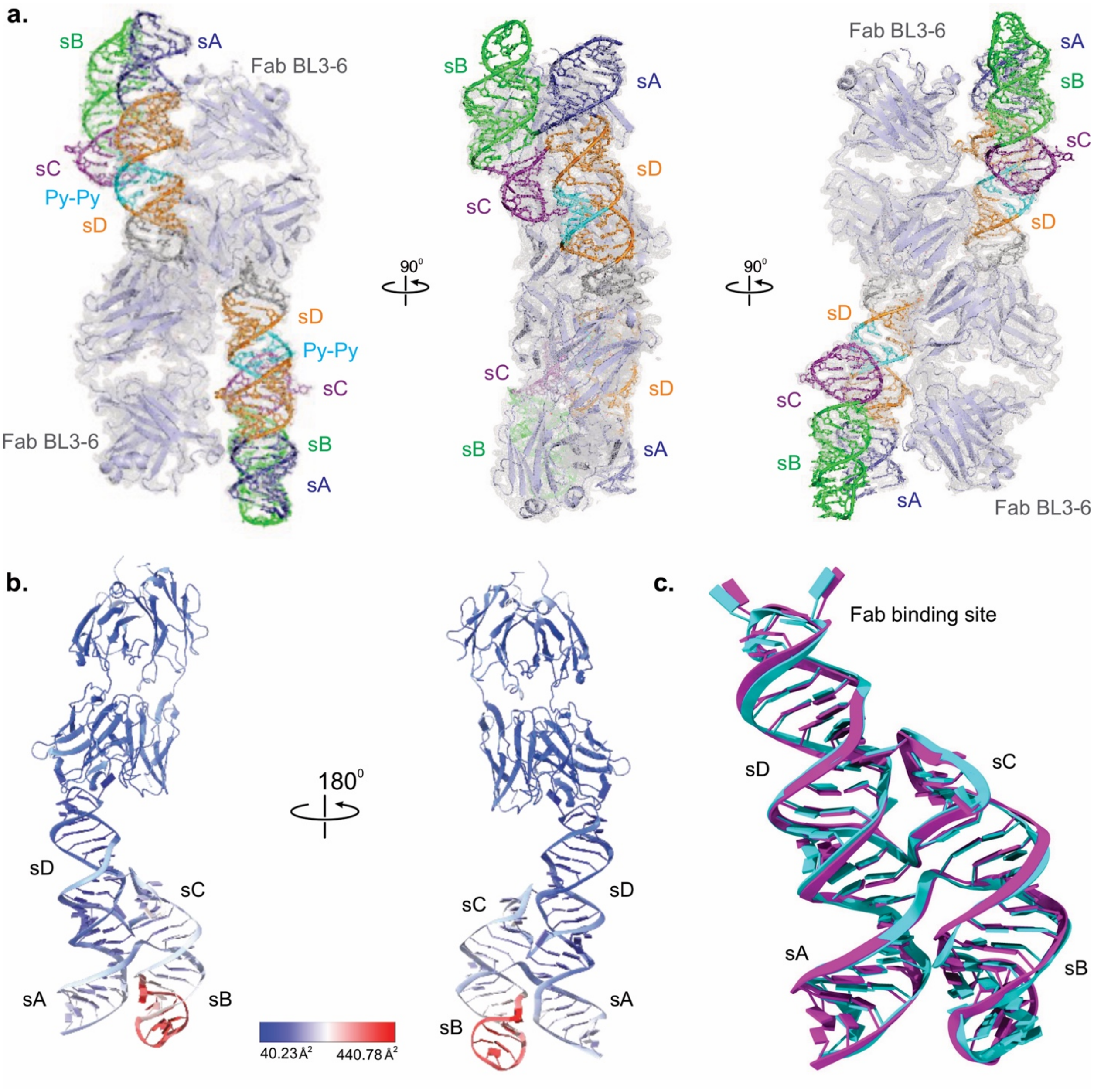

Supplementary Figure S9: Overall crystal structure of the RVC15-DL$_{mut}$ REPLR construct. (a) The crystal structure of the RVC15-DL$_{mut}$ in complex with Fab BL3-6 solved at 2.54 Å resolution (PDB: 8VMA). The crystals have two Fab-RNA complexes per asymmetric unit. The gray mesh represents the $2|F_o| - |F_c|$ electron density map at 1σ contour level and carve radius 1.8 Å, and the red spheres depict water molecules. (b) The crystal structure of the same complex colored according to the crystallographic B-factors, with blue for the lowest (40.23 Å^2) and red for the highest (440.78 Å^2) B-factors. (c) the superposition of the two RVC15-DL$_{mut}$ molecules within the asymmetric unit (RMSD = 1.025 Å). The two molecules are colored cyan and magenta for clarity.

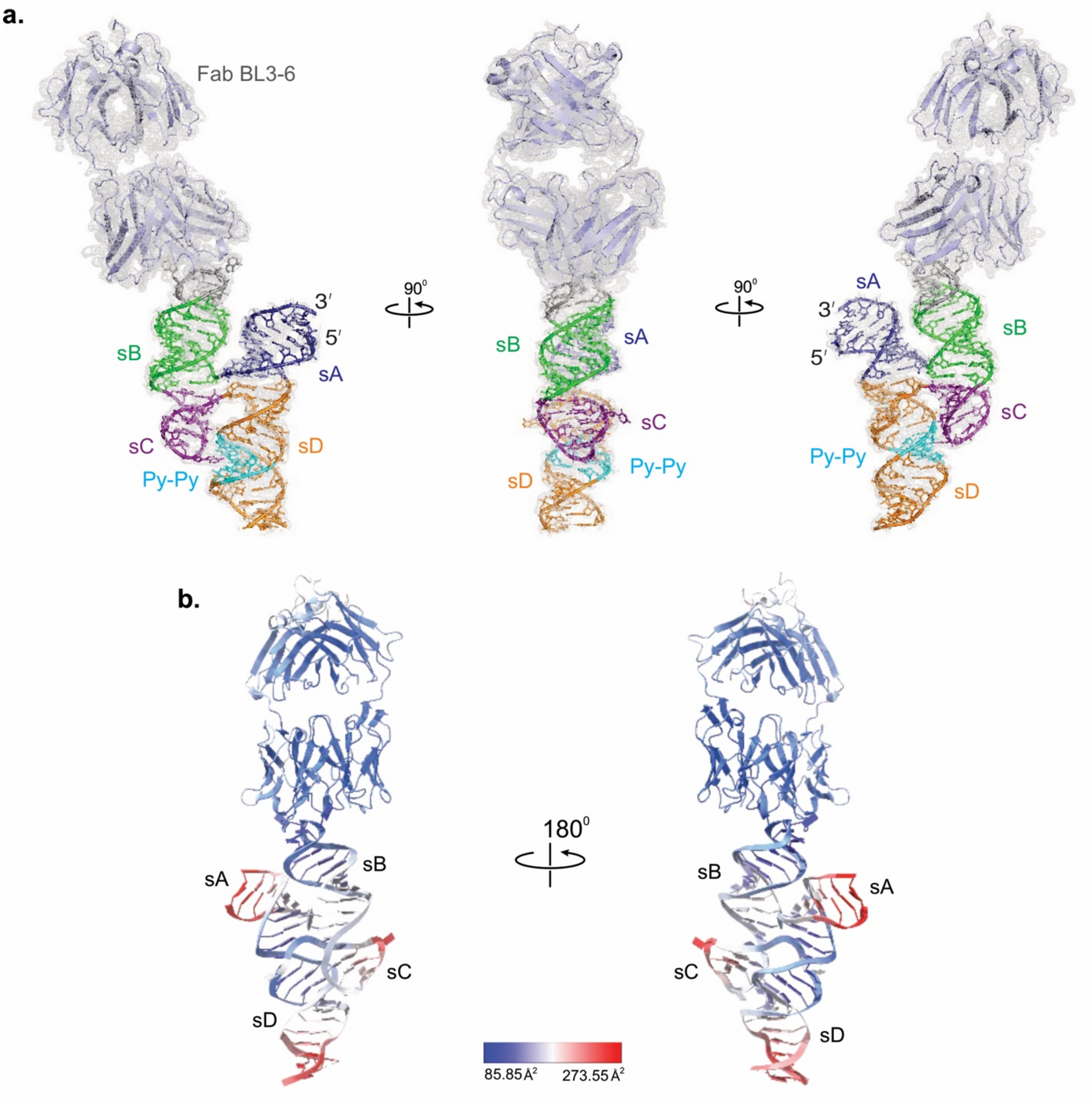

Supplementary Figure S10: Overall crystal structure of the RVC15-BL$_{mut}$ REPLR construct. (a) The crystal structure of the RVC15-BL$_{mut}$ in complex with Fab BL3-6 solved at 2.97 Å resolution (PDB: 8VMB). The crystals have two Fab-RNA complexes per asymmetric unit. The gray mesh represents the $2|F_o| - |F_c|$ electron density map at 1σ contour level and carve radius 1.8 Å. (b) The crystal structure of the same complex colored according to the crystallographic B-factors, with blue for the lowest (85.85 Å^2) and red for the highest (273.55 Å^2) B-factors.

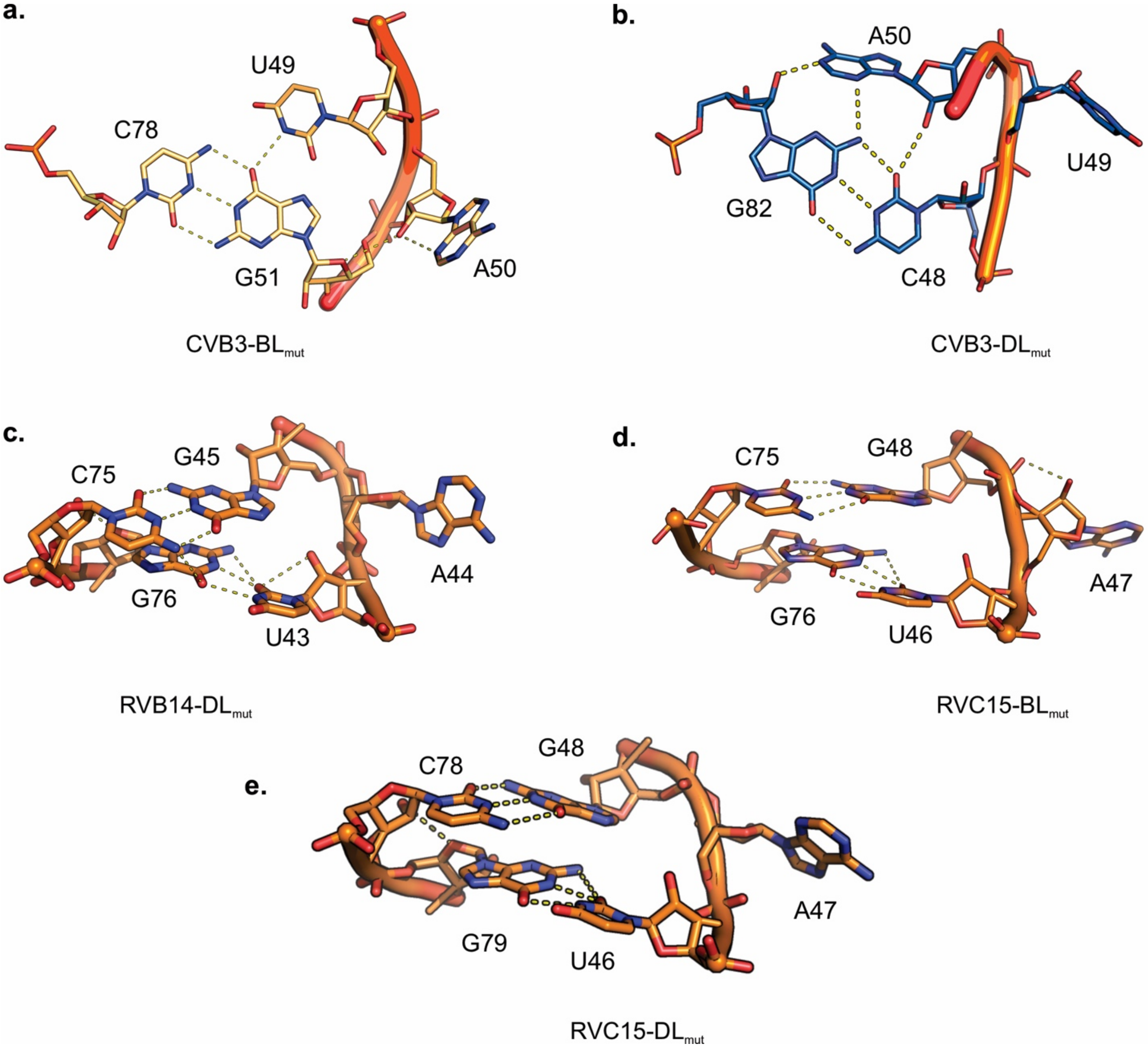

Supplementary Figure S11: The structure of sD-bulge among enteroviral REPLRs. (a) The sD-bulge (a) with the flipped-out A50 as observed in CVB3-DL_{mut} crystal structure and (b) with the filled-out U49 as observed in CVB3-BL_{mut} crystal structure. A single nucleotide bulge with flipped-out (c) A44 as observed in RVB14-DL_{mut}, (d) A47 as observed in RVC15-BL_{mut}, and (e) A47 as observed in RVC15-DL_{mut} crystal structures. The yellow dashed lines represent the distances between the heteroatoms (≤ 3Å) with potential for hydrogen bonding.

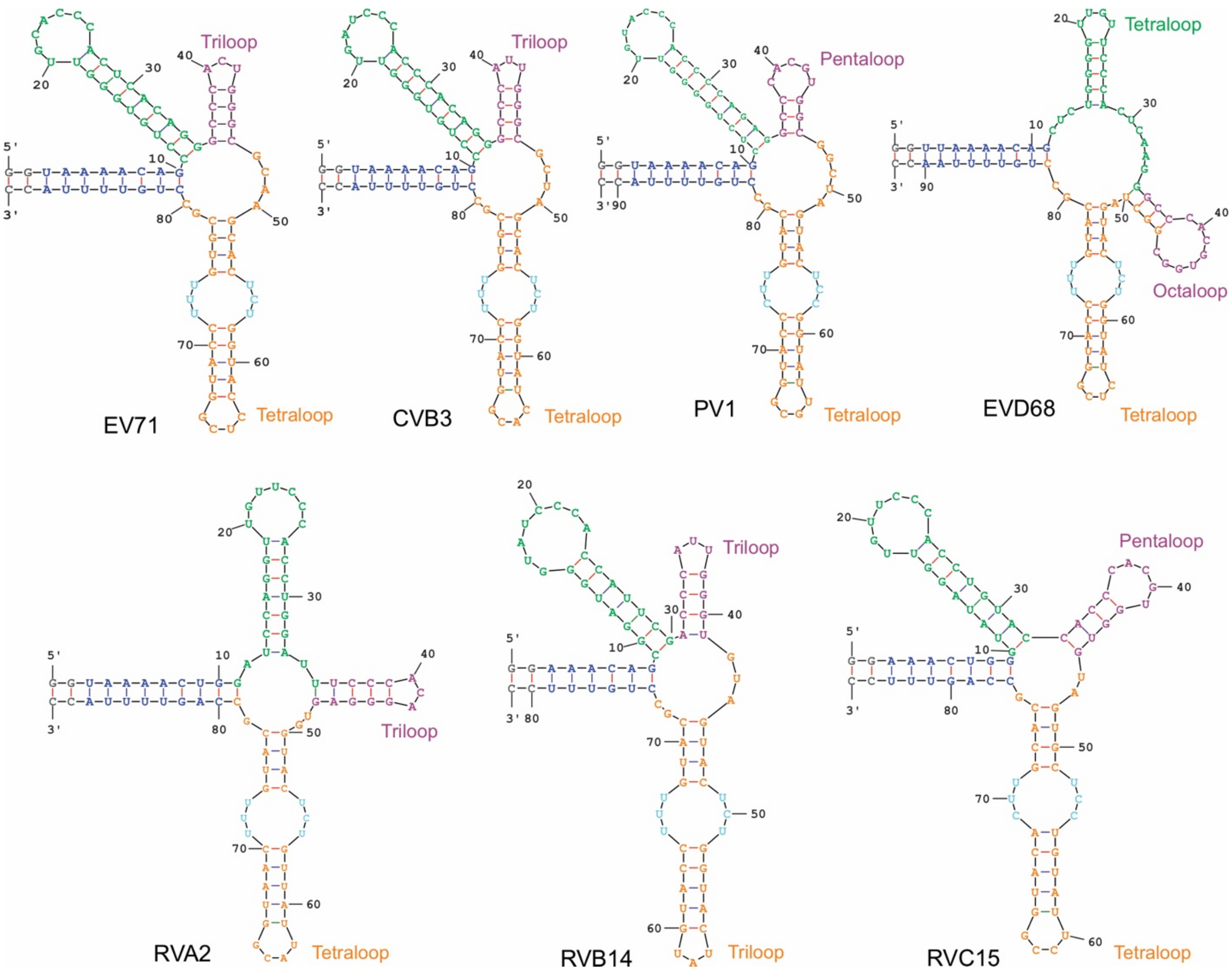

Supplementary Figure S12: The mFold (3) predicted secondary structures of seven enteroviral REPLRs, each representing a species from enterovirus A–D and Rhinovirus A–C. The nucleotides are colored analogously as the corresponding crystal-derived (or homology modeling-derived) secondary structures (see Supplementary Figure S13 below). All structures resemble the crystal-derived (or homology modeling-derived) secondary structures except the EVD68 REPLR. The variations in the sB, sC, and sD loops are indicated.

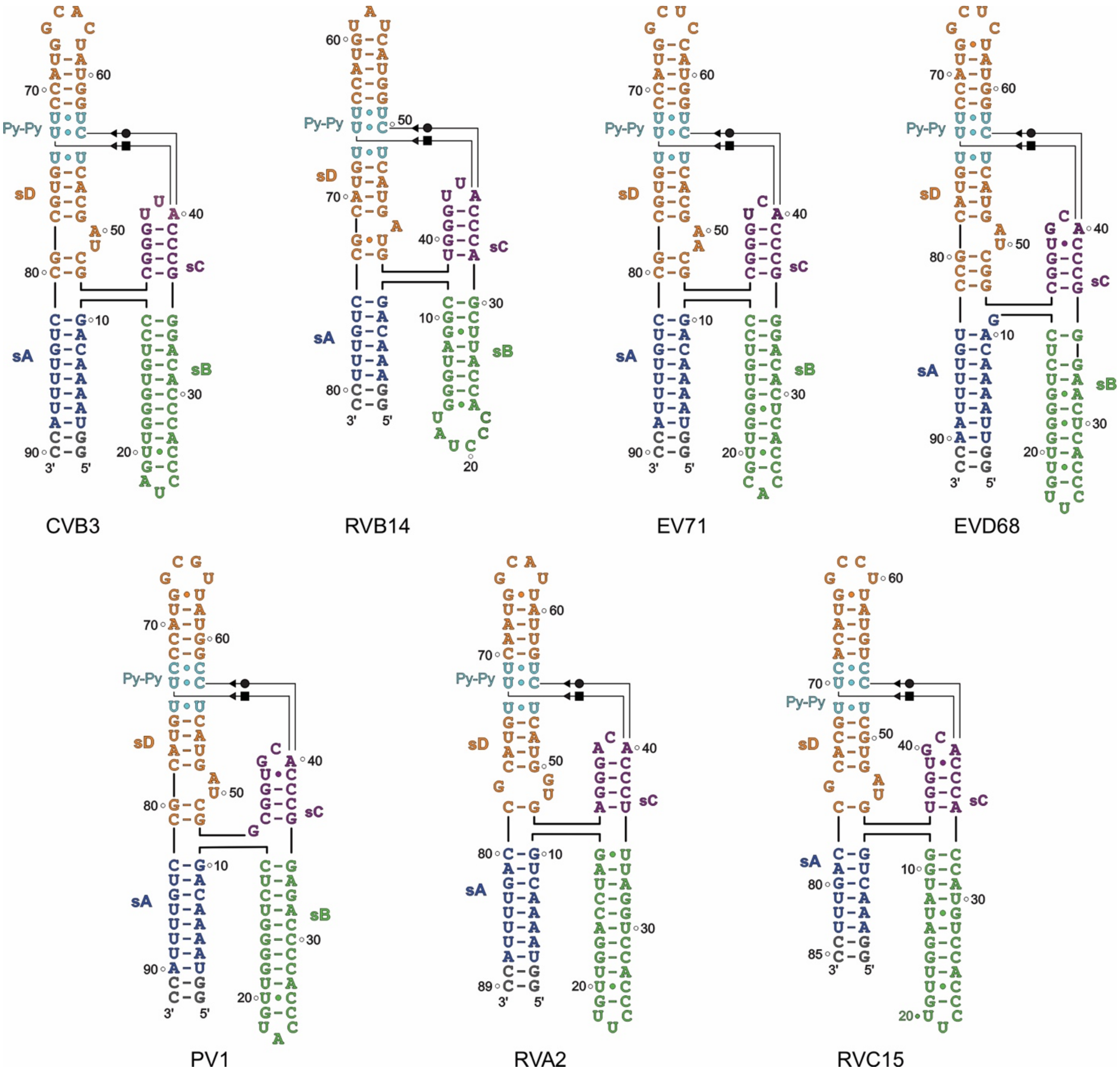

Supplementary Figure S13: Structures of seven enteroviral REPLRs, each representing a species from enterovirus A–D and Rhinovirus A–C. The secondary structures shown are derived from the lowest-energy model predicted by homology modeling using A•C•U base-triple as a template. These predicted models for the CVB3, RVB14, RVC15, and PV1 (4) are topologically similar to their corresponding crystal structures.

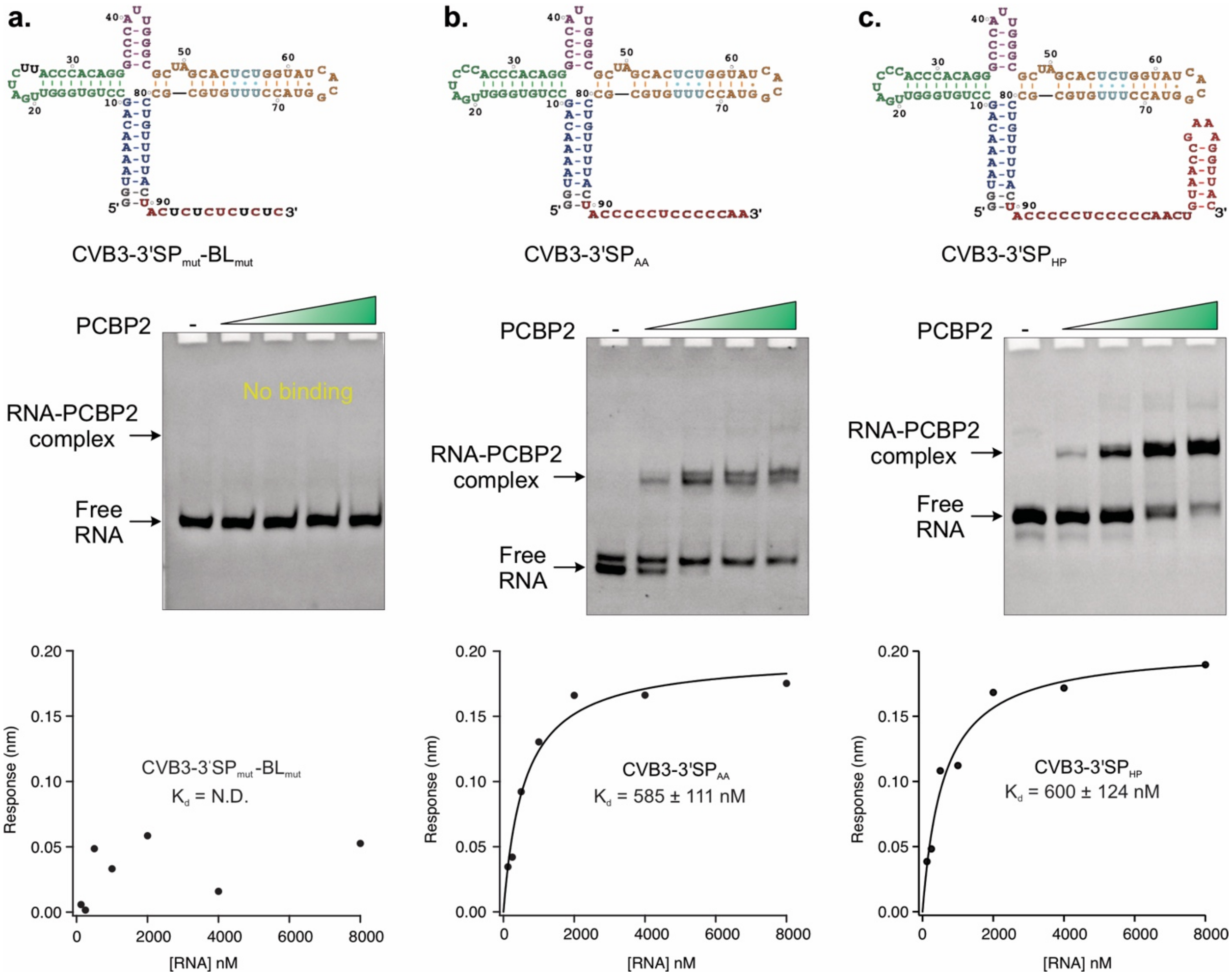

Supplementary Figure S14: The CVB3 REPLR 3′SP constructs and PCBP2 binding. (a) The CVB3-3′SP_{mut}-BL_{mut} construct (top panel), an nPAGE (middle panel) and the BLI measurements (bottom panel) show no binding with human PCBP2. (b) The CVB3-3′SP_{AA} construct (top panel), an nPAGE (middle panel) and the BLI measurements (bottom panel) show similar binding of the PCBP2 as the CVB3-3′SP (see Figure 4b,c). The two RNA bands suggest misfolded conformation that inhibits the PCBP2 binding, as only the lower RNA band shows a dose-dependent response for the PCBP2. (c) The CVB3-3′SP_{HP} construct (top panel), an nPAGE (middle panel) and the BLI measurements (bottom panel) show no influence of the extended hairpin sequence on the PCBP2 binding. Each gel lane was loaded with 100 ng (300 nM) of RNA. The green color-gradient triangle shows the PCBP2 concentration range from 0.3 – 1.2 μM. The filled circles in binding isotherms represent the BLI responses for a given RNA concentration, and the solid curve depicts the fitting (see methods for details). N.D. depicts not determined, and the reported Kd values are the mean ± standard deviation (n = 3).

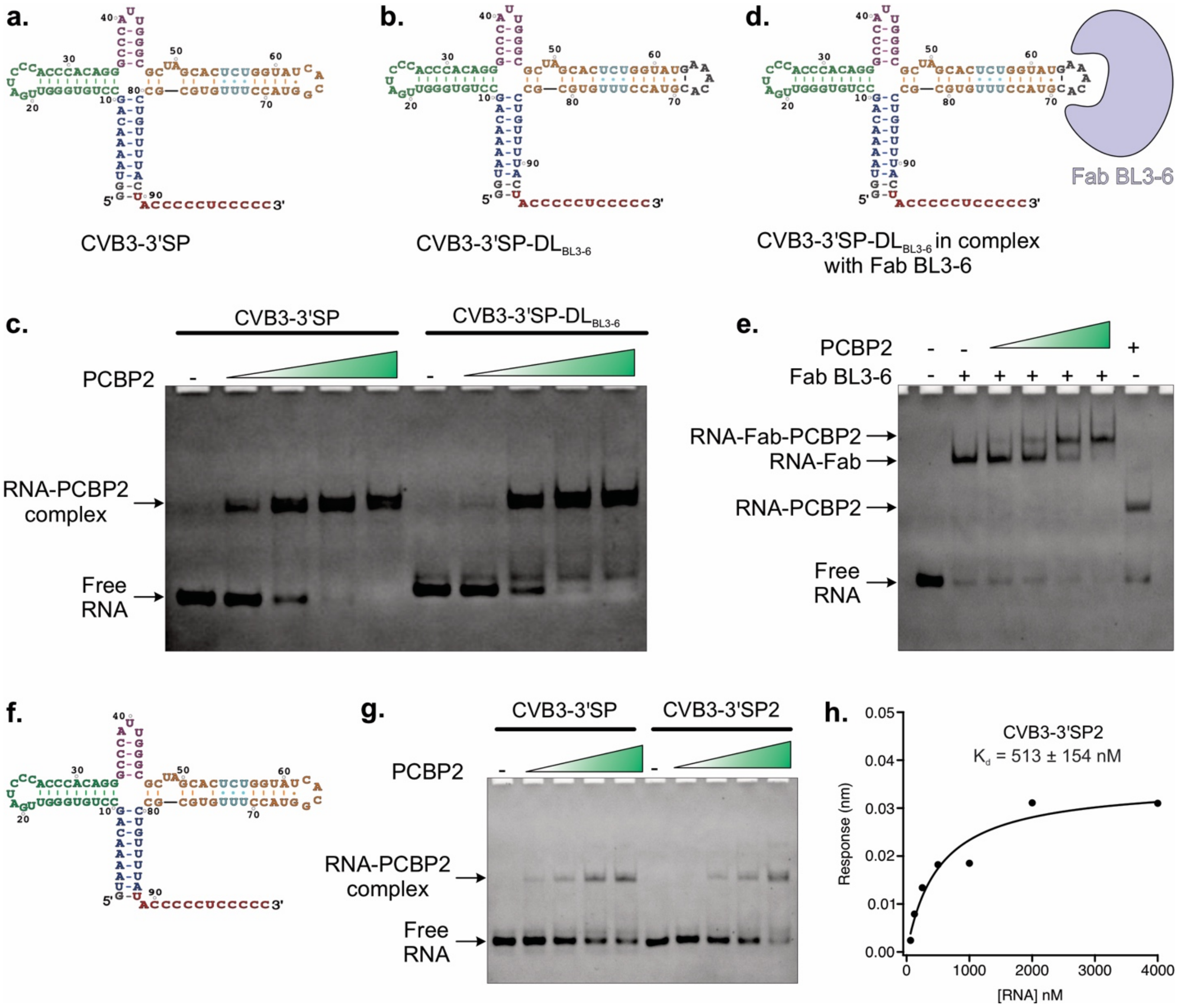

Supplementary Figure S15: The CVB3 REPLR constructs with the 3′SP sequences and PCBP2 binding in the presence of Fab BL3-6. The nPAGE analysis (c) for the constructs CVB3-3′SP (a) and (b) CVB3-3′SP-DL$_{BL3-6}$ shows that grafting of the Fab-binding sequence in the sD-loop does not influence PCBP2 binding. The binding of the Fab BL3-6 (e) with the CVB3-3′SP-DL$_{BL3-6}$ construct (d) does not affect the PCBP2 binding, suggesting that the sD and sB loops are distal to each other with no long-range interactions between them. The CVB3-3′SP2 construct (f) without the sA stem stabilization by G-C pairs as in our crystallization constructs (see supplementary Figure S3) showed similar binding as the CVB3-3′SP in nPAGE (g) and BLI assays (h), indicating that such stabilization has no significant impact on the REPLR structure and the PCBP2 binding.

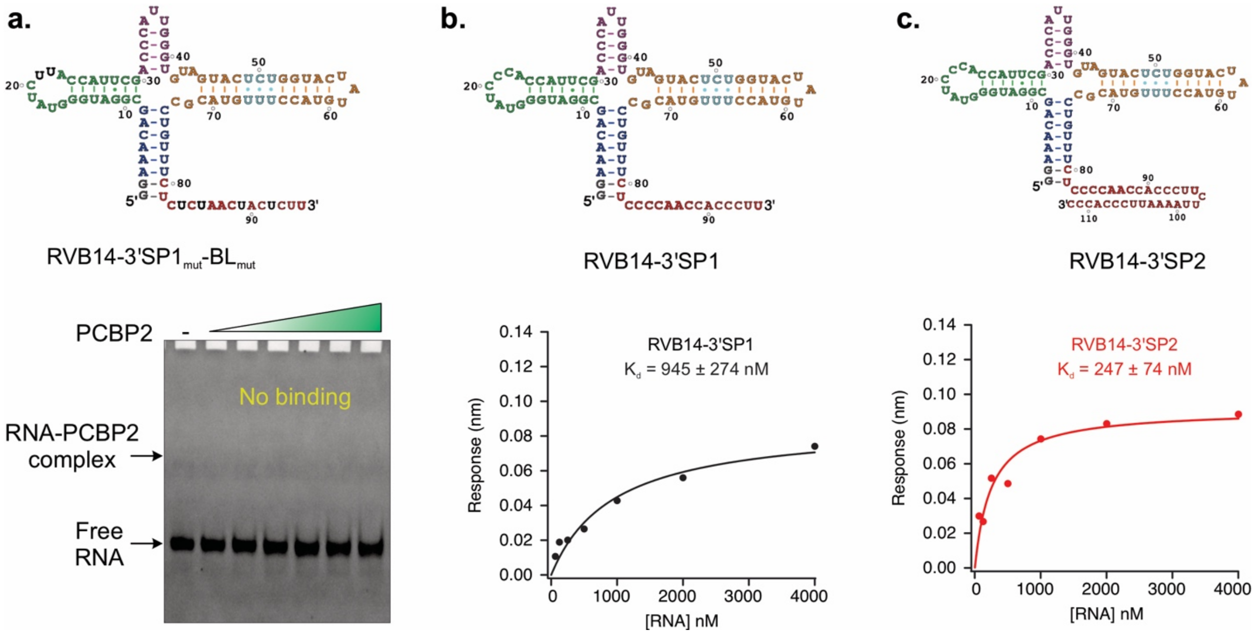

Supplementary Figure S16: The RVB14 REPLR 3′SP constructs and PCBP2 binding. (a) The RVB14-3′SP1$_{mut}$-BL$_{mut}$ construct (top panel) and an nPAGE (bottom panel) show no binding with human PCBP2. (b) The RVB14-3′SP1 construct (top panel) and the BLI-based binding isotherm for its interactions with the PCBP2 (bottom panel). (c) The RVB14-3′SP2 construct (top panel) and the BLI-based binding isotherm for its interactions with the PCBP2 (bottom panel). Each gel lane was loaded with 100 ng of RNA. The filled circles in binding isotherms represent the BLI responses for a given RNA concentration, and the solid curve depicts the fitting (see methods for details). N.D. depicts not determined, and the reported K_d values are the mean ± standard deviation (n = 3).

Supplementary Information References

1. Bailey, J.M. and Tapprich, W.E. (2007) Structure of the 5' nontranslated region of the coxsackievirus B3 genome: Chemical modification and comparative sequence analysis. *J Virol*, **81**, 650-668.
2. Das, N.K., Hollmann, N.M., Vogt, J., Sevdalis, S.E., Banna, H.A., Ojha, M. and Koirala, D. (2023) Crystal structure of a highly conserved enteroviral 5′ cloverleaf RNA replication element. *Nature Communications*, **14**, 1955.
3. Zuker, M. (2003) Mfold web server for nucleic acid folding and hybridization prediction. *Nucleic Acids Research*, **31**, 3406-3415.
4. Gottipati, K., McNeme, S.C., Tipo, J., White, M.A. and Choi, Kyung H. (2023) Structural basis for cloverleaf RNA-initiated viral genome replication. *Nucleic Acids Research*, **51**, 8850-8863.
